# Accuracy of consumer-level and research-grade activity trackers in ambulatory settings in older adults

**DOI:** 10.1371/journal.pone.0216891

**Published:** 2019-05-21

**Authors:** Salvatore Tedesco, Marco Sica, Andrea Ancillao, Suzanne Timmons, John Barton, Brendan O’Flynn

**Affiliations:** 1 Tyndall National Institute, University College Cork, Cork, Ireland; 2 Centre for Gerontology and Rehabilitation, University College Cork, Cork, Ireland; Baylor College of Medicine, UNITED STATES

## Abstract

Wrist-worn activity trackers have experienced a tremendous growth lately and studies on the accuracy of mainstream trackers used by older adults are needed. This study explores the performance of six trackers (Fitbit Charge2, Garmin VivoSmart HR+, Philips Health Watch, Withings Pulse Ox, ActiGraph GT9X-BT, Omron HJ-72OITC) for estimating: steps, travelled distance, and heart-rate measurements for a cohort of older adults. Eighteen older adults completed a structured protocol involving walking tasks, simulated household activities, and sedentary activities. Less standardized activities were also included, such as: dusting, using a walking aid, or playing cards, in order to simulate real-life scenarios. Wrist-mounted and chest/waist-mounted devices were used. Gold-standards included treadmill, ECG-based chest strap, direct observation or video recording according to the activity and parameter. Every tracker showed a decreasing accuracy with slower walking speed, which resulted in a significant step under-counting. A large mean absolute percentage error (MAPE) was found for every monitor at slower walking speeds with the lowest reported MAPE at 2 km/h being 7.78%, increasing to 20.88% at 1.5 km/h, and 44.53% at 1 km/h. During household activities, the MAPE climbing up/down-stairs ranged from 8.38–19.3% and 10.06–19.01% (dominant and non-dominant arm), respectively. Waist-worn devices showed a more uniform performance. However, unstructured activities (e.g. dusting, playing cards), and using a walking aid represent a challenge for all wrist-worn trackers as evidenced by large MAPE (> 57.66% for dusting, > 67.32% when using a walking aid). Poor performance in travelled distance estimation was also evident during walking at low speeds and climbing up/down-stairs (MAPE > 71.44% and > 48.3%, respectively). Regarding heart-rate measurement, there was no significant difference (p-values > 0.05) in accuracy between trackers placed on the dominant or non-dominant arm. Concordant with existing literature, while the mean error was limited (between -3.57 bpm and 4.21 bpm), a single heart-rate measurement could be underestimated up to 30 beats-per-minute.

This study showed a number of limitations of consumer-level wrist-based activity trackers for older adults. Therefore caution is required when used, in healthcare or in research settings, to measure activity in older adults.

## Background

The availability and use of wrist-worn activity trackers and smartwatches have experienced a tremendous growth in the last 5 years, with new brands and models released every year claiming improved features and capabilities. The user-friendliness of these wearable devices has promoted device location at the wrist versus other body locations [1].

Typical smartwatches contain several sensors (accelerometer, gyroscope, magnetometer, barometer/altimeter, GPS, heart rate etc.) and are designed with enough battery life to last for weeks or even months depending on the sensor modalities integrated in the system [2]. Thus, these devices find applications in a wide range of fields, from sport and fitness to entertainment and healthcare [3].

In healthcare, their main goal is to gather information in order to define and evaluate the wearer’s physical activity and health status across certain parameters, in real-time and in temporal summation, including the potential to predict the person’s future health status. Common consumer-level, wrist-worn devices typically provide data on steps, distance travelled, physical activity, energy expenditure, and sleep pattern. Moreover, these wearable platforms may represent an excellent tool for encouraging a healthier lifestyle by promoting activities, and working to influence behavior through user-friendly visual feedback of current health status [4]. As per a review of devices in 2018 [5], out of the brands currently available, the five most used trackers in medical research projects were the Fitbit, Garmin, Misfit, Apple, and Polar, with the Fitbit being the most commonly used. Only a few well-established brands of trackers have been thoroughly validated, although there is a growing research and clinical interest in the validity and reliability of those trackers [6].

Steps are a major indicator of the wearer’s physical activity. Achieving 10,000 steps daily is recommended for positively influencing body composition (weight, body mass index, body fat, etc.) and improving health parameters, such as heart health and quality of life [7]. However, the accuracy of step counting devices is controversial, and while some studies found a good reliability of step counting, [8], others have questioned their accuracy, even in ambulatory settings [9]. In particular, fitness trackers may provide acceptable results for walking at normal speed, or for brisk/vigorous walking, but can be highly inaccurate for slower walking speeds (< 2 km/h) [10–12]. Moreover, it has been reported that particular use conditions such as outdoor use, using a walking aid, or performing daily activities (e.g. eating), may negatively impact on the step counting performance [13–14], and consequently, reduce their reliability in real-world scenarios and health promotion programs [15].

Another important health indicator measured by wrist-worn devices is heart rate. Again, results on the accuracy of commercial devices are not overall sufficient and are highly dependent on the brand considered, and thus require further investigations. For example, Stahl et al. [16] observed that several activity trackers had a limited MAPE (between 3.3% and 6.2%) during both walking and running activities. Similarly, Sartor et al. [17] demonstrated that a wrist-worn optical device (Philips Electronics OHRM [17]) performed acceptably close to the gold-standard (which was an ECG-based chest strap Polar H3) in a broad range of activities in a heterogeneous, healthy population, and showed initial promising results also in patients with heart disease. On the other hand, Benedetto et al. [18] observed in a Fitbit Charge 2 (Fitbit Inc., USA) that even though the mean bias was modest, the individual heart rate measure could plausibly be underestimated by almost 30 bpm. Moreover, it has been discovered that heart rate measurements in Fitbit trackers may be affected by significant errors in free-living conditions [19], especially in moderate-to-vigorous physical activity heart rate ranges. Heart rate and physical activity are generally related to energy expenditure indicators, which are provided by several commercial brands. However, no consumer-level device is yet known to deliver accurate energy expenditure estimations in ambulatory settings, with large discrepancies between monitors [20–21].

In summary, at present mainstream devices show good capability to measure heart rate, number of steps, distance, and sleep duration, but the measurement accuracy of energy consumption is still inadequate, with validity and reliability in free-living situations still representing a challenge [22–27].

However, all the studies mentioned above were conducted on young or middle-aged adults, mostly in good health. Considering the multiple applications of wrist-based technology and the potential adoption in healthcare, and cognizant of an aging population, it is important to investigate the use of these devices in different populations, such as older people [28]. Research has explored the acceptability of commercial trackers to older adults [29–30], showing the current barriers to acceptability and the limitations of the available devices (in terms of data accessibility, ease-of-use, and wearability being impacted by the effects of ageing on skin, such as wrinkling and dryness of the skin, and so on), and defining the features that future older population-oriented trackers should provide to address acceptability issues [30]. Though older adults perceive trackers as useful and acceptable, with the devices providing positive health outcomes in physician-led wellbeing programs [31], the market for older person-specific activity trackers is still in its infancy [30]. Few studies have investigated the validity of mainstream wrist-based activity trackers in healthy older adults, and most of these were mainly related to step-counting.

For example, Burton et al. [32] reported a good reliability and validity for the Fitbit Flex and Fitbit Charge HR, even though both devices underestimated step count within the laboratory environment during a 2-minute walking test (2MWT). On the other hand, Phillips et al. [33] determined that a Fitbit worn on the wrist underestimated true steps and that good accuracy was mostly achieved for gait speeds > 2 km/h. Finally, Floegel et al. [34] observed that the Jawbone UP appeared accurate at measuring steps in older adults with normal and impaired ambulation during a self-paced walking test, but was not accurate at measuring steps in cane-users, concluding that other positions on the body (e.g. hip or ankles) may be more suitable when monitoring steps in older adults with varied gait patterns.

In other work [35], the caloric expenditure of a Fitbit (worn on the waist) showed good correlation with a self-report CHAMPS (Community Healthy Activities Model Program for Seniors) physical activity questionnaire for older adults.

Recently, Straiton et al. [36] reviewed the validity and reliability of consumer-grade activity trackers in older community-dwelling adults, considering seven observational studies, most of them with small sample sizes, which, however, mainly considered measurements of step count and duration of physical activity, proving that slow walking and impaired ambulation impact devices’ performance.

Given the importance that physical activity has on older adults in order to improve cardiorespiratory and muscular fitness, bone and functional health, and reduce the risk of depression and cognitive decline, activity trackers may be beneficial in promoting an increase in physical activity in older adults. However, it is evident that a comprehensive comparative analysis of currently available, mainstream trackers worn by healthy older people in walking and in non-walking scenarios which goes beyond a simple step counting evaluation is still needed. The present study aims to investigate the validity of different activity trackers in the estimation of: step count; distance walked; and heart rate; across a number of walking/household/sedentary activities recreated in a lab-environment in a cohort of older adults.

## Methods

### Participants

The study described in this publication is based on a sample of 18 healthy older people (7 males, 11 females) aged between 65 and 74. Volunteers were recruited via a general invitation e-mail, posters, and word of mouth, to staff and ex-staff at University College Cork, and also through local social and voluntary groups that had older adults as members, who were informed of the study by the Centre for Gerontology and Rehabilitation in University College Cork.

The inclusion criteria were age > = 65 years, with no history of neurological or other disorders or disability that could affect subject’s movements, and in good general health.

Prior to participation, volunteers received a verbal and written explanation of the study protocol and written consent was obtained. Socio-demographic information was collected on gender, age, weight, height, and dominant arm. The study received approval by the Clinical Research Ethics Committee at the University College Cork. All participants gave written consent prior to commencement in the study.

### Equipment

The following consumer-level/research-grade devices were selected for evaluation. Details are illustrated in Table 1.

**Table 1:** Device characteristics.

	Fitbit Charge2	Garmin VivoSmart HR+	Philips Health Watch	Withings PulseOx	ActiGraph GT9X-BT	Omron HJ-72OITC	Polar H7
**Internal sensor**	3-axis accelerometer Heart monitor Altimeter	3-axis accelerometer Heart-rate monitor Altimeter GPS	3-axis accelerometer Heart-rate monitor	3-axis accelerometer Heart-rate monitor Altimeter SpO2 sensor	3-axis accelerometer 3-axis gyroscope 3-axis magnetometer	2-axis accelerometer	Heart-rate monitor
**Output**	Step Distance Floor climbing Calories Heart rate Physical activity intensity Activity recognition Sleep analysis	Step Distance Floor climbing Calories Heart rate Physical activity intensity Activity recognition Sleep analysis	Step Calories Heart rate Respiration rate Physical activity intensity Activity recognition Sleep analysis	Step Distance Elevation Calories Heart rate Running SpO2 level Sleep analysis	Step Energy expenditure METs Physical activity intensity Sleep analysis Raw data	Step Distance Calories Physical activity intensity	Heart rate
**Communication**	Bluetooth	Bluetooth, ANT+	Bluetooth	Bluetooth	Bluetooth, USB	USB	Bluetooth
**Memory size**	Up to 7 days	Up to 7 days	Up to 7 days	NA	4 GB	35 days	NA
**Battery life**	Up to 5 days	GPS mode: 8 hs Smart mode: 5 days	Up to 4 days	Up to 14 days	About 14 days	6 months	150 hours
**Size**	Width: 21.4 mm Thickness: 12.7 mm Small circ: 14–17 cm Large circ: 17–20.6 cm XL cir: 20.6–23.6 cm	Width: 21 mm Thickness: 15 mm Regular circ: 13.6–19.2 cm XL cir: 18–22.4 cm	Width: 36 mm Thickness: 12 mm Small circ: 13.7–18.9 cm Large cir: 16.7–22.3 cm	Width: 22 mm Thickness: 8 mm Height: 43 mm	Width: 35 mm Thickness: 10 mm Height: 35 mm	Width: 47 mm Thickness: 20 mm Height: 71 mm	Width: 35 mm Thickness: 10 mm Height: 64 mm
**Weight**	34 g	Regular: 31 g X-large: 33 g	NA	8 g	14 g	NA	25 g
**Attachment site**	Wrist	Wrist	Wrist	Wrist, waist	Hip, wrist, ankle, thigh	Waist	Chest
**Display screen**	Yes	Yes	Yes	Yes	Yes	Yes	No
**Cost**	Approx 120 €	Approx 200 €	Approx 250 €	Approx 80 €	Approx. 400 €	Approx 50 €	Approx 90 €

**Fitbit Charge 2** (Fitbit Inc., San Francisco, CA, USA). A wrist-based device with large OLED screen featuring heart rate monitoring, tracking of steps, distance, calories burned, floors climbed, active minutes, and sleep duration.

**Garmin VivoSmart HR+** (Garmin, Olathe, Kansas, USA). A wrist-based device that monitors heart rate, calories burned, intensity of fitness activities, distance, time and pace for indoor or outdoor activities.

**Philips Health Watch** (DL8791, Philips, Stamford, CT, USA). A wrist-worn device which monitors heart rate, heart rate zones, and resting respiration rate. It can track steps, active minutes and activities, such as walking, biking or running. The device is water-resistant and with a battery lifetime up to 4 days.

**Withings Pulse Ox** (Withings, Paris, France). A wrist-worn device which can measure heart rate, sleep patterns, steps taken, calories burned, elevation climbed, and distance travelled. The Pulse Ox also measures blood oxygen level to assess the overall efficiency of the respiratory system.

**ActiGraph GT9X-BT** (ActiGraph LLC, Pensacola, FL, USA). A research-grade activity monitor. The device includes a 3-axis gyroscope, a magnetometer and an accelerometer and can provide raw data and a variety of objective activity and sleep measures (including activity counts, energy expenditure, steps taken, activity/sedentary bouts, sleep latency/efficiency, etc.) using publicly available validated algorithms. Sampling frequency for inertial data is up to 100 Hz. Battery life is 14 days and the device can store up to 4 GB. The device can be worn on the waist, hip, wrist, ankle, or thigh.

**Omron HJ-72OITC** (Omron Healthcare, Kyoto, Japan). The device is worn on the hip and is a full-featured pedometer (steps, distance, calories). The pedometer is based on a two-axis design. The replaceable battery has a 6 month lifespan with normal use. The device has no reset button or on/off switch, it is always on and it resets itself to zero at midnight daily and keeps previous daily totals in memory.

**Polar H7** (Polar OY, Kempele, Finland). A gold-standard chest-worn heart rate sensor which can transmit data up to 5 kHz via BLE. It is water-proof up to 30 m, and battery lifetime up to 150 h (operation time). ECG is obtained via electrodes embedded in the textile strap which is kept firmly in place. The device has been validated [37] and was thus used as a gold-standard for heart rate measurements.

In the present investigation, we took into account indicators related to step-counting, distance travelled, and heart rate as common to all the devices visualization interfaces.

The devices considered for evaluation were chosen after carrying out an international literature review which identified the most diffused commercial devices to be the Fitbit and Garmin (later confirmed by Henriksen et al. [5] as being included in 58 out of 61 validation studies available on MEDLINE) and ActiGraph, Polar H7 and Omron HJ-72OITC as additional research-grade trackers/pedometers. At the time of the data collection, Fitbit Charge 2 and Garmin VivoSmart HR+ were the most innovative products of the Fitbit and Garmin brands.

Finally, as also indicated in the acknowledgments, this study was part of the EU H2020 project ProACT, which includes as partners also Philips and the Dundalk Institute of Technology which, in their research agenda also include the Philips Health Watch and the Withings Pulse Ox, respectively. Thus, it was decided to include also those two devices in the final evaluation.

### Experimental protocol

In the chosen cohort, all the wrist-worn brands were tested, along with the additional pedometer, trackers, and heart rate sensors located on the hip, chest and ankle. During every activity the participant was wearing 2 trackers on both wrists (4 overall), with a pair of devices of the same model worn simultaneously on the dominant and non-dominant arm for comparison. Every activity was repeated twice in order to test all the trackers while wearing only a maximum of two wrist-worn devices at the same time so as to minimize any possible interference between the devices. Similar considerations were also previously taken into account in literature; for example, authors in [32] considered a GENEActiv accelerometer worn on the wrist together with 2 Fitbit Flex or Fitbit Charge, for overall three devices on the same wrist. It was indicated that the level of agreement between the same devices was excellent, suggesting then that the positioning of the devices was not significantly affecting the performance. Likewise, Floegel et al. [34] adopted on the same wrist a Fitbit Flex along with a Jawbone UP. The two ActiGraph monitors were located on the dominant waist (midaxillary line) and dominant ankle, as those locations are reported to be optimal for monitoring step count in older adults [38–39]. Step-counts from the ActiGraph were obtained offline using the only algorithm implemented on the ActiLife software (ActiGraph LLC., Pensacola, FL, USA).

**Fig 1:**
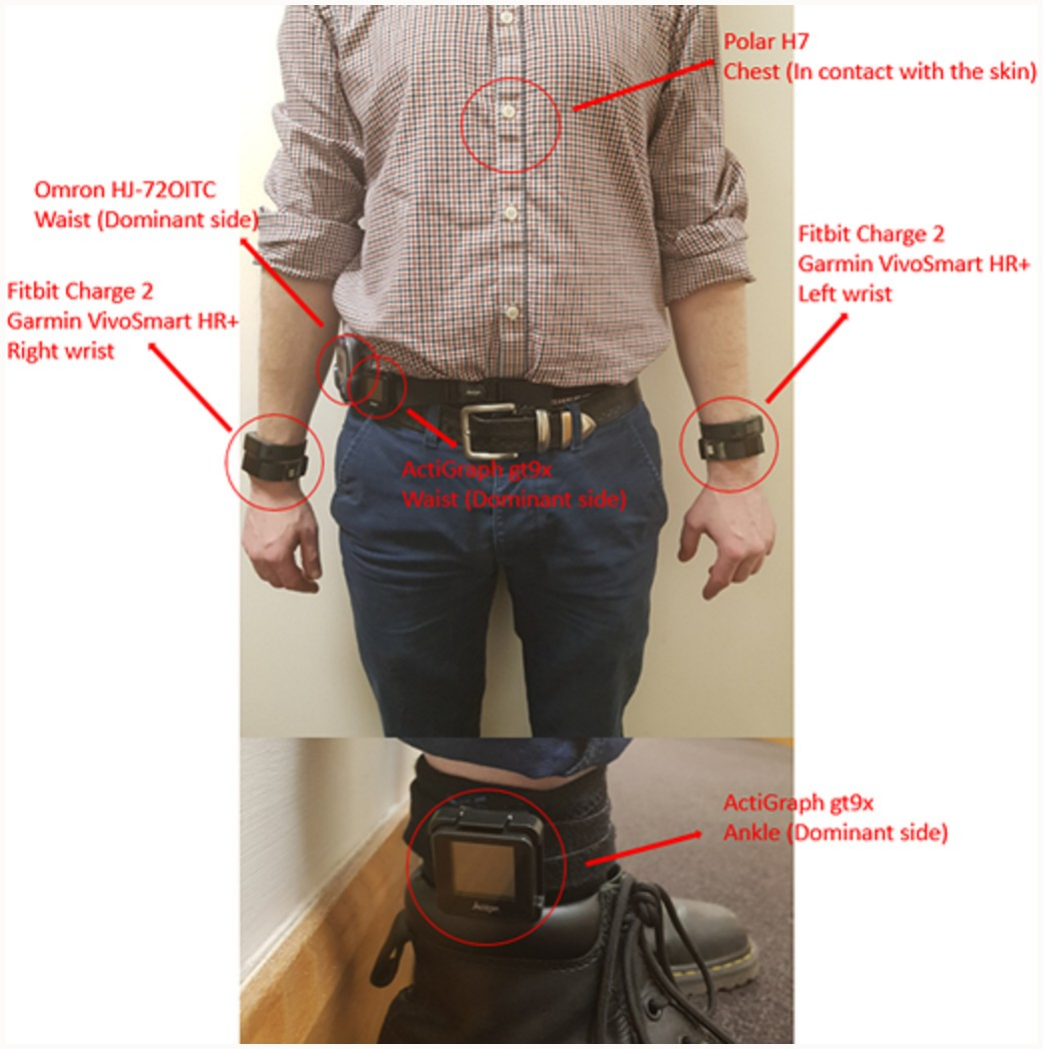
Devices positioning. Polar H7, Fitbit Charge 2, Garmin VivoSmart HR+, ActiGraph GT9X-BT, Omron HJ-72OITC. Philips Health Watch and Withings Pulse Ox are not illustrated in the picture but are worn in the same position of the Fitbit and Garmin devices.

The Omron device was worn on the hip, while the Polar H7 product was worn on the chest. Fig 1 shows the placement of the devices on one participant.

The order of testing for the trackers in a subject, the position of the trackers on the wrists for each activity (i.e. nearer to, or further from, the hand), and the order of the tasks and activities within a test session were randomized, using a computer-generated sequence. All testing was performed with a subject in one session lasting 3 hours. The volunteers could rest as long as they required between the exercises to avoid fatigue. Participants were requested to perform activities to allow analysis of specific biometric parameters during three different scenarios: walking on a treadmill, household activities, and sedentary activities.

In the first scenario, participants were asked to walk on a treadmill at 1, 1.5 and 2 km/h for 3 min. Subjects were asked to walk without holding on with their hands.

The chosen household activities were:

- walking while carrying a box (for 3 min),
- dusting (for 3 min),
- walking with a rollator (for 3 min),
- walking up two flights of stairs, with 10 steps per flight, and back down again.

For the sedentary scenario, the selected activities were writing, reading, and playing cards, again for 3 min each.

Using a walking aid was selected to represent some older adults’ real-world condition. The overall aim was to cover the typical household and sedentary activities performed as part of general activities of daily living.

Step-counting was obtained by all activity trackers, distance by only Fitbit Charge 2, Garmin VivoSmart HR+, and Withings Pulse Ox, and heart rate by only Fitbit Charge 2, Garmin VivoSmart HR+, and Philips Health Watch. The heart rate was measured immediately after the performed activity, within 1 minute of the end of the activity, for all trackers. A similar methodology was also implemented in [22].

Steps were measured during all activities with direct visualization and/or video-recording as reference. Distance was obtained for the treadmill scenario and some household activities (e.g. climb up/down-stairs and walking with a box). The references were, respectively, the treadmill itself and the following two approaches:

1. for the climb up/down-stairs using Eq (1), as discussed in [40]
  $$Distance\;=\;\sqrt{{stairs\_depth}^{2}+{stairs\_height}^{2}}∙number\_stairs+\;\pi ∙r∙number\_turning$$ (1)
  where r is defined as the turning radius for each flight of stairs.
2. For the walking with a box experiment, distance was calculated by multiplying the number of steps visually observed (via direct visualization) by the average stride length, which is estimated before the test and obtained for each volunteer by measuring the travelled distance after 10 steps and dividing it by 10.

Heart rate was measured during the treadmill and the household activities, as those scenarios are associated with higher intensity levels compared to the sedentary activities, and in literature it is reported that this is the Beat-Per-Minute range with the lower HR monitoring validity [41].

### Statistical analysis

Descriptive statistics were run on the computed parameters. The following error indicators were computed for each activity/parameter/device: mean bias with related standard deviation (SD), MPE (Mean Percentage Error) with related SD, MAPE (Mean Absolute Percentage Error), MAD (Median Absolute Deviation), MAE (Mean Absolute Error), and RMSE (Root Mean Square Error). ICC (2,1) (Intra-class correlation) was performed for each tracker compared against the actual values for each participant. ICC, with related 95% confidence intervals (C.I.) was computed as well. ICC values less than 0.5, between 0.5 and 0.75, between 0.75 and 0.9, and greater than 0.90 are indicative of poor, moderate, good, and excellent reliability, respectively. Statistical tests and Bland-Altman plots were also computed. Shapiro-Wilk tests were implemented to test the normality assumption of the data distributions, and as a result, t-test and Wilcoxon signed-rank test were adopted for normally distributed and non-normally distributed data, respectively. Bland-Altman plots were obtained considering the single device against the criterion, and additionally devices from the same brand against each other (i.e. Fitbit worn on the dominant arm against the Fitbit worn on the non-dominant arm). Due to the large number of overall Bland-Altman plots obtained, the final results are not shown graphically but are summarized in a tabular form. All statistical analyses were performed using Matlab (The Mathworks, Natick, MA, USA).

## Results

Overall, eighteen participants took part in the data collection. Characteristics of the participants are illustrated in Table 2. All the subjects were white-skinned of Irish and British ancestry. Data collection was carried out at the Tyndall National Institute between October 2017 and March 2018. Results shown in Tables A-L in S1 File are available in the additional files, together with the raw collected data located on an Excel file in the supplementary files.

**Table 2:** Subjects characteristics.

Older Adults		
Number of subjects: 18 (7 males / 11 females)		
***Males***		
	Age (years):	69 ± 3.2
	Height (cm):	175.8 ± 4.8
	Weight (Kg):	79.7 ± 4.46
	People with right dominant arm:	7
	People with left dominant arm:	0
***Females***		
	Age (years):	69.7 ± 2.4
	Height (cm):	162.9 ± 5.8
	Weight (Kg):	66.1 ± 4.25
	People with right dominant arm:	9
	People with left dominant arm:	2

One participant (Subject 12) did not perform the treadmill tasks properly as she felt uncomfortable without using the handrails and the data has not been considered. Also, some data points are missing due to lack of device synchronization, device malfunctioning, etc., and those data are reported as an “X” in the Excel file. As a result, 11.6% of the overall data points have been discarded for these reasons.

### Steps

#### Walking test

The step-counting performance for the treadmill test is presented in Table A in S1 File. At 1 km/h, all the wrist-worn devices significantly underestimated steps. The worst performance was obtained with the Withings Pulse Ox with a MAPE of 86.07% (dominant arm) and 86.98% (non-dominant arm), while Fitbit Charge 2 showed the best performance with a MAPE of 44.53% and 44.86% on both arms, which also resulted in the best RMSE of 119 steps. Waist-worn devices, such as ActiGraph and Omron, also performed poorly. ICC obtained against gold-standard was poor (< 0.5) for every tracker, except for ActiGraph on the ankle which showed a moderate agreement. P-values were < 0.01 for all trackers when tested against the reference.

Walking at 1.5 km/h showed similar results. Steps were under-counted for all trackers except Fitbit Charge 2. MAPE was less, ranging from 20.88% (Philips Health Watch non dominant) to 81.55% (Withing Pulse Ox non dominant). The highest RMSE was 196.58 steps and the lowest was 67.82 steps. Omron and ActiGraph (on the waist and the ankle) showed better results compared to 1 km/h. ICC was poor except for Garmin VivoSmart HR+ and ActiGraph on the ankle which showed moderate reliability. Again, all trackers showed p-values < 0.01 except Fitbit Charge 2 and Philips Health Watch.

The devices performed better when walking speed was 2 km/h. The best MAPE were 7.78% and 9.99% for Garmin VivoSmart HR+ on the dominant/non-dominant arm. ICC was poor for Fitbit Charge 2, Withings Pulse Ox, Philips Health Watch (dominant), Omron, and ActiGraph on the waist, it was moderate for Philips Health Watch (non-dominant arm), good for ActiGraph on the ankle, and good-to-excellent for Garmin VivoSmart HR+ (dominant arm ICC: 0.91, 95% CI: 0.28–0.98, non-dominant arm ICC: 0.79, 95% CI: 0.45–0.92). Again, all trackers showed p-values < 0.01 except Fitbit Charge 2 and Philips Health Watch.

#### Household activities

The step-counting performance for the household activities is presented in Table B in S1 File. During the walking upstairs activity, most of the trackers showed comparable characteristics. Among the wrist-based devices, Fitbit Charge 2 showed the best results, with MAPE equal to 8.38% and 10.06%. However, better performances were displayed by non-wrist devices such as Omron (MAPE 7.44%), ActiGraph on the ankle (MAPE 5.81%), and ActiGraph on the waist (MAPE 11.87%). Withings Pulse Ox and Philips Health Watch showed the worst performance among the wrist-worn devices.

Regarding the walking downstairs activity, all trackers generally under-counted steps (MPE between -30.37 and -3.08%). Omron and ActiGraph on the waist were the best performers (RMSE 3.98 and 6.68 steps respectively) while Withings Pulse Ox (on the dominant arm) was the best wristband. The performance of the wrist-worn trackers was comparable and generally similar to the results obtained for the walking upstairs activity. ICC was poor for all trackers except Omron.

In the ‘carry a box test’, the Fitbit Charge 2 device demonstrated the best accuracy, with a MAPE equal to 3.14% and 3.41% (dominant and non-dominant arm), followed by ActiGraph on the ankle (5.36%). ICC was poor for most of the trackers, with Omron and ActiGraph on the ankle showing moderate reliability, and Fitbit Charge 2 having excellent agreement.

Dusting produced significantly different results between trackers worn on the dominant and non-dominant arm (p-values > 0.05 for trackers on the dominant arm, and < 0.012 for trackers on the non-dominant arm). MAPE was always lower on the dominant arm, with the best results reported by Garmin VivoSmart HR+ and Philips Health Watch (57.66% and 58.75%), while the best MAPE on wrist-worn devices on the non-dominant arm was shown by Philips Health Watch (67.19%). Worse results were obtained for Omron and ActiGraph (both on the waist and the ankle) with p-values < 0.01. The lowest RMSE is 63.42 steps. ICC was poor for every tracker.

Finally, all trackers significantly under-counted steps when wearers walked with a rollator walking aid (p-values < 0.01). The lowest RMSE were reported by ActiGraph on the ankle (102.44 steps) and Omron (124.28 steps) which also showed the lowest MAPE. Regarding wrist-based devices, only Fitbit Charge 2 showed MAPE results lower than 80%.

#### Sedentary activities

**Fig 2:**
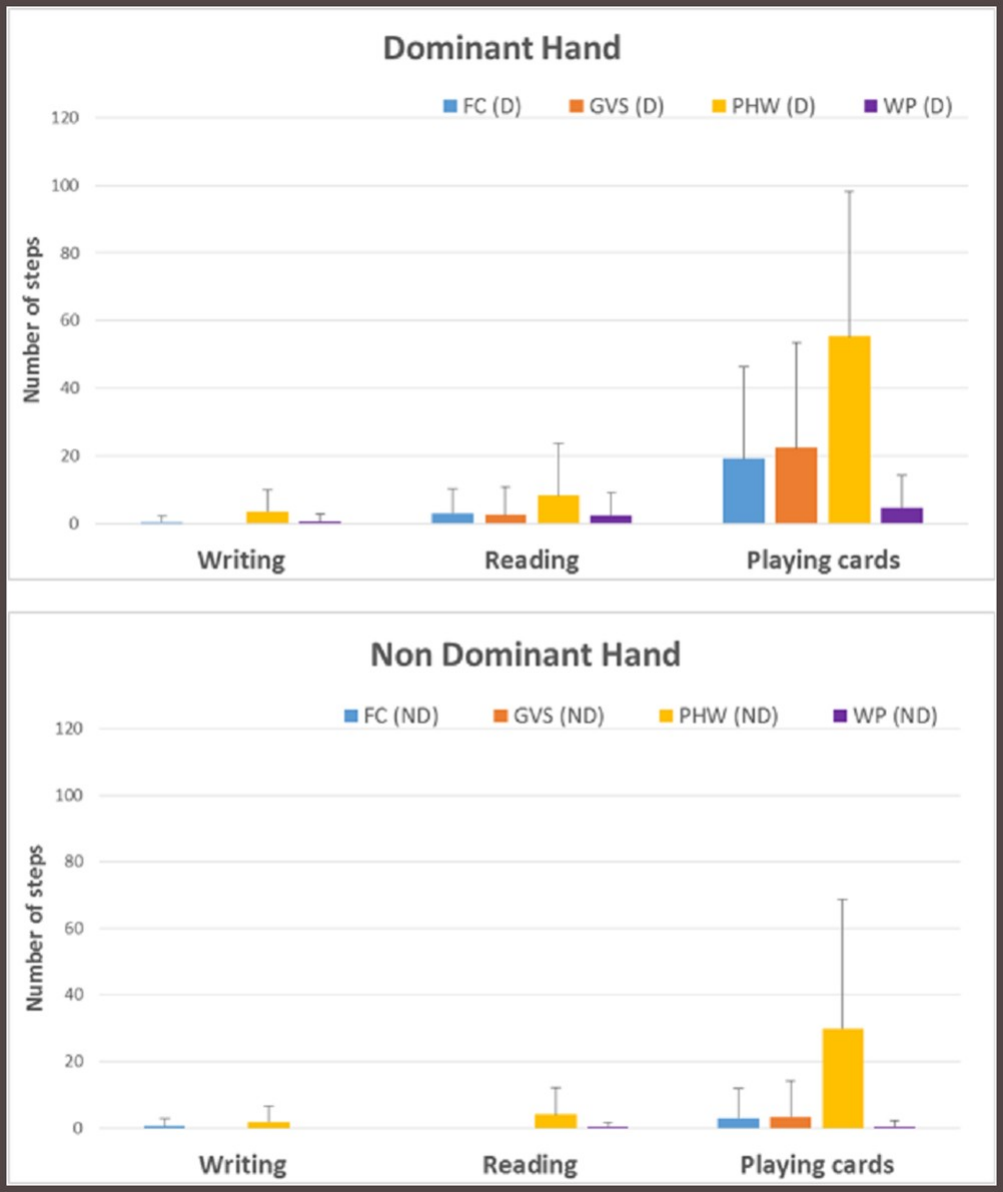
Sedentary activities results—steps. FC: Fitbit Charge 2, GVS: Garmin VivoSmart HR+, PHW: Philips Health Watch, WP: Withings Pulse Ox, D: Dominant, ND: Non Dominant.

Results for the sedentary activities are shown in Fig 2. As those activities were performed in a seated position, only requiring the use of upper extremities, no step was identified during the data collection by video-recording and direct observation. However, with the devices on the dominant arm, some steps were counted by all trackers in every activity, with card playing showing the largest number of false steps. Again Philips Health Watch showed the worst performance as compared to other trackers. The results obtained from the non-dominant arm showed similar performance with a reduced number of false steps. The Philips Health Watch worn on the non-dominant arm also counted significantly more steps compared to other trackers in all scenarios.

### Distance

#### Walking test

The performance for treadmill activities is presented in Table C in S1 File. All trackers over-estimated the travelled distance at every speed, except Withings Pulse Ox. Withings Pulse Ox consistently had the best performance at every speed, with MAPE values for both arms steadily decreasing when increasing speed (112.94% and 123.76% at 1 km/h, 89.25% and 94.9% at 1.5 km/h, 77.5% and 71.44% at 2 km/h). A similar trend towards better performance at higher speeds was evident also for Garmin VivoSmart HR+ and only partially for Fitbit Charge 2. ICC was poor for all activities and trackers.

#### Household activities

The performance for household activities is presented in Table D in S1 File. All trackers significantly over-counted the travelled distance when walking up/down-stairs. Fitbit Charge 2 showed the best MAPE for both arms, at 60.17% / 66.1% (upstairs), and 48.3% / 57.17% (downstairs). ICC was poor for both activities and all trackers. P-values were always < 0.01 for all trackers.

Better results were obtained for the ‘carry a box test’, with Fitbit Charge 2 still showing the best results. Overall, the MAPE was between 6.21% and 5.8%. Fitbit Charge 2 and Withings Pulse Ox under-estimated the travelled distance while Garmin VivoSmart HR+ over-estimated. ICC was poor for Garmin VivoSmart HR+, while was moderate for Fitbit Charge 2 and Withings Pulse Ox. Except for the Garmin VivoSmart HR+ worn on the non-dominant arm, the p-values for all the trackers were > 0.05.

### Heart rate

#### Walking test

The performance related to heart rate measurements for treadmill activities are presented in Table E in S1 File. ICC shows moderate-to-good reliability for all trackers at 1 and 1.5 km/h, while it was moderate-to-excellent at 2 km/h. Monitor devices have comparable performance at every speed, with RMSE in the range between 8.82 and 10.71 bpm (at 1 km/h), 8.42 and 12.16 bpm (at 1.5 km/h), and 5.69 and 15.53 bpm (at 2 km/h). The worst-case MAPE was equal to 13.89%, with the best one being 4.54%. p-values were > 0.05 for all trackers and every speed.

#### Household activities

The performance related to heart rate measurements for household activities are presented in Table F in S1 File. ICC shows good-to-excellent reliability for all trackers and every activity. Monitor devices have comparable outcomes, with the worst RMSE being 12.45 bpm (Fitbit Charge 2 non dominant) and the best RMSE equal to 3.76 bpm (Fitbit Charge 2 dominant). The worst-case MAPE was 9.42%, with the best one being 3.57%. Again, p-values were > 0.05 for all trackers and every activity.

## Discussion

The present investigation assessed and compared the accuracy of consumer-level and research-grade activity trackers (mostly wrist-worn devices, but also waist-worn, and ankle-worn) against gold-standards in a number of ambulatory activities in an ageing population.

The walking scenario in our study provided useful insights on trackers’ performance at different speeds.

At walking speeds of 1–2 km/h, every tracker, including both wrist-based and non wrist-based devices, had decreasing accuracy with decreasing walking speeds, which resulted in a significant steps under-counting. Performances were similar among the wrist-based monitors (except for the Withings Pulse Ox), and also there was no significant difference between devices on the dominant and non-dominant arm (with the exception of the Philips Health Watch at 1 km/h). A large MAPE was displayed by every monitor at all these speeds (1 km/h, 1.5 km/h and 2 km/h); for instance, the lowest reported MAPE at 2 km/h was 7.78%, while it increased to 20.88% at 1.5 km/h, and 44.53% at 1 km/h. Waist-worn devices also showed large MAPE values, which were lower on the ankle-worn device indicating that this position may be more suitable for detecting steps in slow-walker (MAPE: 8.87% at 2 km/h). These findings confirm results in literature [36] which indicate that in general lower speeds might be challenging for any device for accurate step detection. Since older people and hospitalized people tend to walk at very slow (< 1.5 km/h) or slow speeds (between 1.5 and 3 km/h), and since walking represents the most common physical activity performed by the ageing population, our results question the adoption of consumer-level trackers for step detection in an ageing cohort.

Regarding the household activity category, the MAPE reported for climbing up/down-stairs ranged from 8.38–19.3% and 10.06–19.01%, respectively, for dominant and non-dominant arm. On the other hand, waist-based devices had more uniform performance in these activities. As a consequence, wrist-based trackers’ performance in those activities may provide less reliable results in an ageing cohort compared to other body positions.

In contrast to other activities, the ‘carry a box’ test showed no particular difference between wrist-worn and non wrist-worn devices.

Dusting was selected to emulate more complex real-life activities in a lab setting. Non wrist-based devices showed poor performance. The best MAPE was shown by the ActiGraph device which was worn on the waist, at 89.81% and which significantly under-counted steps. Wrist-worn devices, on the other hand, showed particular differences between dominant and non-dominant arms. The range of MAPE for the dominant arm was 57.66–66.88%, while for the non-dominant arm this was 67.19–95.98%. It is evident that monitors should be worn on the dominant arm for a performance improvement when performing such unstructured activities.

The mean bias of the sensor readings taken when wearing the device on the dominant arm tends to be lower compared to when being worn on the non-dominant arm, however, the related standard deviation behaved in the opposite way. As a consequence, the 95% limits of agreement are reduced in results obtained on the non-dominant arm. For instance, the best reported 95% limits of agreement for the dominant arm was (-87.9, 146.1) steps, and (-132.7, -42) steps for non-dominant arm. This difference between arms is likely due to the fact that only one arm is typically adopted during those activities (usually the dominant arm) and, as a consequence, the continuous movements of the arm may be counted as steps, therefore reducing the overall error. It is thus evident that unstructured activities represent a serious challenge for activity trackers regardless of the location on the body. These results confirm the findings reported in [42] and are not unexpected given the common assumption that physical activity equates to moderate paced or brisk walking, or jogging, whereas for a frailer older person, physical activity is mainly through activities of daily living, such as meal preparation and light housework [43].

Finally, the rollator activity showed that all wrist-based trackers significantly under-count steps, with the best MAPE being 67.32% (Fitbit Charge 2). This in unsurprising, given that while walking with a rollator, the arms do not swing, but move en-bloc with the rollator in a slow steady forward movement rather than what a device could detect as a ‘step’. Non wrist-worn sensors performed better, with MAPE ranging between 45.96% (ActiGraph on the ankle) and 58.79% (Omron). These findings confirm results in literature regarding the inaccuracy of commercial-based wrist-worn devices when used with a walking aid [13], and are comparable with the results achieved when performing tasks with stationary upper extremities (i.e. pushing a stroller) as shown in [44]. Significant attention has been recently paid by researchers to overcome the challenge of accurately detecting steps in people who require a walking aid [45] through the adoption of wearable inertial sensors, whose consideration for human motion analysis (e.g. on lower-limbs [46–47], upper-limbs [48], or for activity recognition [49]) is nowadays well-established in literature.

During sedentary activities, discrepancies were evident between dominant and non-dominant arms. Wearing the device on the dominant arm, some steps were counted during all activities, and playing cards led to errors in most trackers, especially Philips Health Watch. There was a similar but lower error related to a device on the non-dominant arm. Thus, wrist-worn devices are not only inaccurate during light household tasks, they also count false steps in sedentary activities, as also shown in [42], and under count steps while walking with a walking aid. Thus, an active but slow-walking older person may appear relatively inactive, while a sedentary older person who plays long card games will paradoxically appear more active.

In regard to the estimation of travelled distance, MAPE ranged from 71.44–100.63% (at 2 km/h), to 89.25–149.17% (at 1.5 km/h), and 112.94–163.53% (at 1 km/h).

Likewise, results among the physical monitors did not show particular discrepancies during the climbing up/down-stairs activity. Nevertheless, the best MAPE displayed was 48.3%.

Poor performance in estimation of travelled distance were evident for walking at low speeds and climbing up/down-stairs and still represents an issue for consumer-level wrist-mounted trackers. Given the importance that travelled distance has for nurses and clinicians (such as the six minute walk test), it is essential to define accurate algorithms for a reliable estimation in different contexts. As an additional recommendation, for use on an older population, travelled distance should be displayed on the trackers in metres and not in tens of metres. Those findings are aligned with outcomes shown for a young cohort in [50–51] where wrist-worn devices provide high MAPE values (from 25% up to 50%) revealing no good validity in covered distance estimation. It is evident that, in an ageing cohort characterized by a slower ambulation, those errors are even more significant.

Regarding the static measurements of heart rate, there was no significant difference between results obtained by all the trackers on the dominant or non-dominant arm. The RMSE was between 3.76 bpm and 15.53 bpm. The best-case 95% limits of agreement was (-8.3, 6.6) bpm while the worst-case was (-30, 32.9) bpm. MAPE values among all the devices is in the range between 3.57% and 13.89%, with an overall accuracy of 7.3%. These results are aligned with previous investigations [22] carried out with a similar methodology on a young cohort, which presented a MAPE for heart rate monitoring between 4% and 12%, with an overall accuracy of 8%. The slight variation in performance in the older people cohort may be due to skin changes with ageing, and/or arterial stiffness with aging, which may affect trackers’ heart rate monitoring performance. Also, these findings largely confirm a previous study [18], and while the mean bias could be limited, an individual’s heart rate measure could plausibly be underestimated by almost 30 bpm. This limitation can be an impediment to the adoption of consumer-level wrist-based sensors for accurate and real-time physiologic parameters monitoring in healthcare applications. Further studies should be considered, including subjects with more varied heart-rates, i.e. subjects with cardiovascular disorders.

A limitation of this study is that it was not conducted in a free-living environment, thus further studies in free-living conditions are recommended. Given the lack of uniform protocols in literature regarding the comparison and evaluation of physical activity monitors, it was decided to evaluate trackers in a steady-state lab-environment. However, to replicate free-living conditions, several unstructured scenarios were included which provide useful insights into consumer-level activity trackers.

This study was limited to healthy older subjects and the results are not generalizable to impaired older adults or hospitalized people. Thus, further studies would be needed to investigate activity trackers’ performance also in non-healthy populations. Also, the sample size considered for evaluation is limited and future studies should consider to perform a complete evaluation on a larger group of participants. Potential interference between devices worn simultaneously on the same wrist might also represent a possible limitation of the study which require further investigation in future studies.

Unfortunately, no continuous heart rate measurements during activities were possible with the selected devices at the current time. Indeed, some trackers required the subjects to be static and immobile for a few seconds for a correct heart rate measurement. Due to this limitation, this investigation considered only one static measurement at the end of the activity. This approach is also due to the fact that most of the trackers can only provide updates every minute. Even though some trackers could provide measurements every second (e.g. Fitbit using the related API), this does not occur for all the trackers. Moreover, the fact that some trackers can only measure while the subject is static and immobile makes the 1-minute recording during the activities not feasible. Therefore, in order to perform a comparative study with the selected wearable trackers, the only possibility was to reduce the measurements to only once every minute with the subjects in a static position. Despite the limitation of this data, the authors do believe this data is worth retaining as collected using a methodology already adopted in [22]. However, future devices may include this feature (for instance, Fitbit have released the Fitbit PurePulse, which is claimed to perform continuous heart rate monitoring, after this investigation was started) and, thus, future more granular studies will be required to assess their accuracy.

## Conclusion

This study explored the performance of several trackers estimating steps, travelled distance, and heart rate measurements in a cohort of subjects ≥65 years. The findings of this study largely confirmed previous results in literature for a number of trackers and health parameters. Different scenarios were considered, including household activities and sedentary activities. In particular, lower speeds might be challenging for any device for accurate step detection and, likewise, unstructured activities or using a walking aid represent a serious challenge for consumer-level activity trackers. Also, while the mean error for the heart rate measurements could be limited, an individual measure could plausibly be significantly underestimated. Our study highlights the current deficits in accuracy of commercial devices if used by an older person performing typical daily activities, suggesting to use them cautiously for research and clinical applications when involving a cohort of older, and slower-walking people.

## Supporting information

S1 File Supporting_Information_Files. (DOCX)

S2 File Raw_Data_Supplementary_file. (XLSX)

## Abbreviations

2MWT: 2-minute walking test
C.I.: confidence intervals
CHAMPS: Community Healthy Activities Model Program for Seniors
ICC: intra-class correlation
MAD: median absolute deviation
MAE: mean absolute error
MAPE: mean absolute percentage error
MPE: mean percentage error
RMSE: root mean square error
SD: standard deviation
