# Supplementary material for: Accuracy of consumer-level and research-grade activity trackers in ambulatory settings in older adults
Source: PLoS One. 2019 May 21;14(5):e0216891. doi: 10.1371/journal.pone.0216891 (PMC6529154; doi:10.1371/journal.pone.0216891)
Supplement: S1 File — (DOCX) [file pone.0216891.s001.docx]

**Table A. Treadmill Test Results – Steps.**

| **TREADMILL** | | **Fitbit (D)** | **Fitbit (ND)** | **Garmin (D)** | **Garmin (ND)** | **Philips (D)** | **Philips (ND)** | **Withings (D)** | **Withings (ND)** | **ActiGraph (A)** | **ActiGraph (W)** | **Omron** |
| --- | --- | --- | --- | --- | --- | --- | --- | --- | --- | --- | --- | --- |
|  |  |  |  |  |  |  |  |  |  |  |  |  |
| **1 Km/h** | **Mean Percentage Error ± St. Dev. (%)** | -43.31 ± 32.13 | -39.46 ± 38.12 | -53.54 ± 36.29 | -53.01 ± 36.01 | -50.67 ± 42.82 | -39.29 ± 48.5 | -86.07 ± 24.18 | -86.98 ± 26.15 | -50.91 ± 33.18 | -87.05 ± 18.58 | -77.53 ± 31.69 |
|  | **MAPE (%)** | 44.53 | 44.86 | 54.74 | 53.01 | 51.30 | 44.76 | 86.07 | 86.98 | 51.20 | 87.05 | 77.60 |
|  | **MAD (Step)** | 34.00 | 44.00 | 90.00 | 101.00 | 112.00 | 143.00 | 0.00 | 0.00 | 78.50 | 14.00 | 7.00 |
|  | **MAE (Step)** | 103.76 | 104.53 | 121.35 | 111.41 | 112.24 | 95.24 | 200.41 | 201.59 | 109.03 | 207.91 | 178.06 |
|  | **RMSE (Step)** | 119.37 | 119.62 | 138.61 | 129.28 | 141.24 | 129.38 | 206.15 | 208.93 | 124.18 | 217.11 | 191.72 |
|  | **ICC (95% CI)** | 0.44(-0.11 0.79) | 0.44(-0.09 0.77) | 0.36(-0.11 0.73) | 0.46(-0.11 0.81) | 0.41(-0.10 0.75) | 0.45(-0.03 0.76) | 0.22(-0.03 0.63) | 0.24(-0.04 0.65) | 0.51(-0.10 0.82) | 0.11(-0.04 0.39) | 0.24(-0.07 0.60) |
|  |  |  |  |  |  |  |  |  |  |  |  |  |
| **1.5 Km/h** | **Mean Percentage Error ± St. Dev. (%)** | 9.97 ± 37.34 | 8.16 ± 36.11 | -21.84 ± 22.82 | -25.63 ± 27.69 | -17.28 ± 38.88 | -10.14 ± 33.16 | -76.2 ± 30.84 | -81.55 ± 26.58 | -31.02 ± 28.66 | -76.37 ± 25.47 | -58.92 ± 34.25 |
|  | **MAPE (%)** | 29.31 | 28.33 | 22.32 | 25.66 | 28.39 | 20.88 | 76.20 | 81.55 | 31.78 | 77.20 | 58.92 |
|  | **MAD (Step)** | 58.00 | 57.50 | 49.00 | 44.00 | 45.50 | 42.50 | 8.00 | 9.00 | 76.00 | 31.50 | 85.00 |
|  | **MAE (Step)** | 67.50 | 69.25 | 52.35 | 58.65 | 66.56 | 51.06 | 181.18 | 188.59 | 71.26 | 186.94 | 140.59 |
|  | **RMSE (Step)** | 85.54 | 86.28 | 67.82 | 81.70 | 97.29 | 85.89 | 195.14 | 196.58 | 91.44 | 193.80 | 162.32 |
|  | **ICC (95% CI)** | 0.30(-0.20 0.68) | 0.41(-0.09 0.74) | 0.64(-0.01 0.88) | 0.58(-0.01 0.85) | 0.44(<-0.01 0.75) | 0.44(-0.02 0.76) | 0.13(-0.06 0.46) | 0.18(-0.04 0.56) | 0.52(-0.05 0.80) | 0.17(-0.05 0.50) | 0.22(-0.09 0.55) |
|  |  |  |  |  |  |  |  |  |  |  |  |  |
| **2 Km/h** | **Mean Percentage Error ± St. Dev. (%)** | 5.11 ± 40.29 | 10.45 ± 35.5 | -7.44 ± 6.2 | -7.74 ± 13.08 | 1.52 ± 31.31 | 4.11 ± 23.61 | -59.54 ± 38.04 | -57.45 ± 34.9 | -7.77 ± 12.61 | -62.53 ± 29.19 | -32.2 ± 31.36 |
|  | **MAPE (%)** | 27.65 | 26.50 | 7.78 | 9.99 | 20.38 | 11.24 | 59.90 | 57.45 | 8.87 | 62.99 | 32.76 |
|  | **MAD (Step)** | 51.00 | 44.00 | 15.00 | 29.50 | 49.00 | 32.00 | 87.00 | 95.50 | 28.00 | 64.00 | 65.00 |
|  | **MAE (Step)** | 70.19 | 68.81 | 19.63 | 25.25 | 48.06 | 25.94 | 156.06 | 144.56 | 22.22 | 161.50 | 86.31 |
|  | **RMSE (Step)** | 95.96 | 91.13 | 23.98 | 38.68 | 71.26 | 49.94 | 181.41 | 165.83 | 35.11 | 176.86 | 119.52 |
|  | **ICC (95% CI)** | 0.13(-0.41 0.59) | 0.12(-0.39 0.57) | 0.91(0.28 0.98) | 0.79(0.45 0.92) | 0.47(-0.04 0.78) | 0.63(0.21 0.85) | 0.17(-0.10 0.52) | 0.26(-0.10 0.65) | 0.83(0.56 0.93) | 0.18(-0.08 0.50) | 0.23(-0.08 0.52) |

**Table B. Household Activities Results – Steps.**

| **HOUSEHOLD ACTIVITIES** | | **Fitbit (D)** | **Fitbit (ND)** | **Garmin (D)** | **Garmin (ND)** | **Philips (D)** | **Philips (ND)** | **Withings (D)** | **Withings (ND)** | **ActiGraph (A)** | **ActiGraph (W)** | **Omron** |
| --- | --- | --- | --- | --- | --- | --- | --- | --- | --- | --- | --- | --- |
|  |  |  |  |  |  |  |  |  |  |  |  |  |
| **Climb up the stairs** | **Mean Percentage Error ± St. Dev. (%)** | -1.13 ± 11.55 | -1.74 ± 11.89 | -5.77 ± 22.36 | -3.81 ± 15.69 | 1.17 ± 27.38 | -0.37 ± 21.59 | -10.16 ± 12.61 | -10.58 ± 22.25 | -3.01 ± 7.34 | -7.81 ± 13.77 | -5.12 ± 9.77 |
|  | **MAPE (%)** | 8.38 | 10.06 | 11.08 | 10.59 | 19.30 | 16.32 | 12.67 | 19.01 | 5.81 | 11.87 | 7.44 |
|  | **MAD (Step)** | 4.00 | 6.00 | 2.00 | 5.00 | 8.00 | 9.00 | 2.00 | 4.00 | 3.00 | 3.00 | 2.00 |
|  | **MAE (Step)** | 4.60 | 5.47 | 6.75 | 5.62 | 9.86 | 8.50 | 6.92 | 10.31 | 3.16 | 6.39 | 4.03 |
|  | **RMSE (Step)** | 6.20 | 6.31 | 12.19 | 8.11 | 13.15 | 10.72 | 8.79 | 12.90 | 4.36 | 8.50 | 5.99 |
|  | **ICC (95% CI)** | 0.02(-0.52 0.53) | 0.29(-0.27 0.69) | <-0.01(-0.54 0.53) | 0.31(-0.27 0.72) | -0.03(-0.61 0.51) | 0.09(-0.50 0.59) | -0.15(-0.41 0.30) | 0.10(-0.36 0.57) | 0.32(-0.01 0.59) | -0.12(-0.36 0.18) | 0.03(-0.24 0.32) |
|  |  |  |  |  |  |  |  |  |  |  |  |  |
| **Go down the stairs** | **Mean Percentage Error ± St. Dev. (%)** | -8.4 ± 12 | -10.48 ± 21.54 | -3.08 ± 16.27 | -3.24 ± 18.44 | -10.18 ± 18.7 | -16.68 ± 14.97 | -8.58 ± 10.2 | -13.12 ± 20.85 | -30.37 ± 49.99 | -9.53 ± 9.12 | -3.94 ± 6.18 |
|  | **MAPE (%)** | 9.17 | 17.81 | 11.04 | 12.64 | 16.16 | 16.68 | 9.04 | 18.16 | 43.30 | 10.90 | 5.88 |
|  | **MAD (Step)** | 3.00 | 7.00 | 4.00 | 3.00 | 5.50 | 4.00 | 4.00 | 5.00 | 22.00 | 3.00 | 3.00 |
|  | **MAE (Step)** | 5.20 | 9.87 | 5.94 | 7.06 | 8.86 | 9.36 | 5.00 | 10.13 | 23.72 | 5.97 | 3.21 |
|  | **RMSE (Step)** | 8.34 | 13.01 | 8.48 | 10.50 | 11.33 | 12.46 | 7.22 | 13.57 | 31.75 | 6.68 | 3.98 |
|  | **ICC (95% CI)** | -0.10(-0.40 0.33) | -0.09(-0.46 0.38) | 0.25(-0.27 0.65) | -0.10(-0.58 0.41) | 0.11(-0.30 0.55) | -0.08(-0.27 0.27 ) | 0.26(-0.13 0.63) | -0.07(-0.39 0.36) | 0.01(-0.23 0.31) | 0.24(-0.09 0.55) | 0.61(0.25 0.81) |
|  |  |  |  |  |  |  |  |  |  |  |  |  |
| **Carry a box** | **Mean Percentage Error ± St. Dev. (%)** | -2.49 ± 3.43 | -2.25 ± 4.2 | 0.87 ± 13.86 | 0.29 ± 14.47 | -0.43 ± 18.95 | 2.76 ± 20.63 | -14.61 ± 11.16 | -12.53 ± 11.77 | -1.81 ± 10.31 | -12.67 ± 17.01 | -2.51 ± 11.27 |
|  | **MAPE (%)** | 3.14 | 3.41 | 6.59 | 7.11 | 10.77 | 10.60 | 14.70 | 12.81 | 5.36 | 16.39 | 6.98 |
|  | **MAD (Step)** | 18.50 | 17.50 | 19.00 | 23.00 | 8.50 | 16.50 | 18.50 | 28.00 | 19.50 | 35.00 | 23.00 |
|  | **MAE (Step)** | 9.89 | 10.89 | 19.24 | 20.53 | 29.22 | 28.61 | 48.43 | 41.43 | 16.00 | 48.81 | 20.20 |
|  | **RMSE (Step)** | 13.88 | 14.84 | 36.61 | 37.43 | 47.21 | 51.25 | 62.71 | 56.54 | 29.10 | 61.60 | 32.06 |
|  | **ICC (95% CI)** | 0.92(0.74 0.97) | 0.91(0.76 0.97) | 0.42(-0.08 0.75) | 0.42(-0.08 0.75) | 0.30(-0.20 0.67) | 0.19(-0.31 0.60) | 0.15(-0.13 0.53) | 0.36(-0.10 0.72) | 0.66(0.42 0.81) | 0.40(<-0.01 0.67) | 0.59(0.32 0.77) |
|  |  |  |  |  |  |  |  |  |  |  |  |  |
| **Dusting** | **Mean Percentage Error ± St. Dev. (%)** | 39.93 ± 114.74 | -79.36 ± 21.01 | -18.01 ± 65.35 | -71.09 ± 29.14 | 39.36 ± 64.89 | -42.13 ± 66.07 | 8.83 ± 97.59 | -95.98 ± 6.33 | -90.34 ± 17.2 | -89.81 ± 14.23 | -91.66 ± 12.24 |
|  | **MAPE (%)** | 64.94 | 79.36 | 57.66 | 72.75 | 58.75 | 67.19 | 66.88 | 95.98 | 90.34 | 89.81 | 91.66 |
|  | **MAD (Step)** | 32.00 | 16.00 | 24.00 | 11.00 | 22.00 | 25.00 | 33.00 | 0.00 | 0.00 | 4.00 | 0.00 |
|  | **MAE (Step)** | 50.35 | 77.06 | 53.80 | 67.40 | 51.12 | 64.00 | 52.60 | 87.40 | 80.58 | 80.64 | 82.27 |
|  | **RMSE (Step)** | 72.73 | 82.59 | 63.42 | 73.69 | 64.82 | 72.90 | 63.78 | 90.21 | 83.96 | 84.23 | 85.38 |
|  | **ICC (95% CI)** | -0.17(-0.57 0.31) | 0.01(-0.04 0.13) | 0.07(-0.42 0.54) | -0.06(-0.11 0.17) | -0.02(-0.38 0.41) | -0.10(-0.34 0.28) | <0.01(<-0.55 0.52) | <-0.01(-0.02 0.06) | 0.02(-0.02 0.11) | 0.01(-0.02 0.06) | 0.01(-0.02 0.07) |
|  |  |  |  |  |  |  |  |  |  |  |  |  |
| **Rollator** | **Mean Percentage Error ± St. Dev. (%)** | -78.96 ± 28.65 | -60.55 ± 52.62 | -99.93 ± 0.3 | -99.91 ± 0.34 | -93.8 ± 6.86 | -94.89 ± 8.63 | -99.83 ± 0.69 | -99.83 ± 0.7 | -45.96 ± 37.67 | -84.38 ± 18.51 | -58.79 ± 34.05 |
|  | **MAPE (%)** | 78.96 | 67.32 | 99.93 | 99.91 | 93.80 | 94.98 | 99.83 | 99.83 | 45.96 | 84.38 | 58.79 |
|  | **MAD (Step)** | 18.00 | 42.00 | 0.00 | 0.00 | 8.00 | 5.50 | 0.00 | 0.00 | 90.00 | 18.00 | 84.00 |
|  | **MAE (Step)** | 159.25 | 129.81 | 219.06 | 214.27 | 191.56 | 193.44 | 212.81 | 212.81 | 83.30 | 170.67 | 111.52 |
|  | **RMSE (Step)** | 172.47 | 142.71 | 221.88 | 211.58 | 197.62 | 191.68 | 216.22 | 205.75 | 102.44 | 173.75 | 124.28 |
|  | **ICC (95% CI)** | 0.01(-0.07 0.11) | 0.20(-0.13 0.56) | <-0.01(-0.01 0.02) | <-0.01(-0.01 0.02) | <0.01(-0.02 0.06) | <0.01(-0.02 0.06) | <0.01(-0.01 0.03) | <0.01(-0.01 0.03) | 0.41(-0.11 0.73) | 0.10(-0.02 0.36) | 0.29(-0.09 0.66) |

**Table C. Treadmill Test Results – Distance.**

| **TREADMILL** | | **Fitbit (D)** | **Fitbit (ND)** | **Garmin (D)** | **Garmin (ND)** | **Withings (D)** | **Withings (ND)** |
| --- | --- | --- | --- | --- | --- | --- | --- |
|  |  |  |  |  |  |  |  |
| **1 Km/h** | **Mean Percentage Error ± St. Dev. (%)** | 110.59 ± 126.12 | 121.18 ± 147.56 | 85.88 ± 156.01 | 100 ± 174.64 | -28.94 ± 139.48 | -29.88 ± 154.66 |
|  | **MAPE (%)** | 141.18 | 163.53 | 144.71 | 156.47 | 112.94 | 123.76 |
|  | **MAD (Km)** | 0.03 | 0.03 | 0.06 | 0.07 | 0.00 | 0.00 |
|  | **MAE (Km)** | 0.07 | 0.08 | 0.07 | 0.08 | 0.06 | 0.06 |
|  | **RMSE (Km)** | 0.08 | 0.09 | 0.09 | 0.10 | 0.07 | 0.08 |
|  | **ICC (95% CI)** | <0.01(-0.23 0.34) | <0.01(-0.25 0.35) | 0(-0.34 0.41) | <-0.01(-0.34 0.41) | 0(-0.48 0.47) | <-0.01(-0.48 0.47) |
|  |  |  |  |  |  |  |  |
| **1.5 Km/h** | **Mean Percentage Error ± St. Dev. (%)** | 145.83 ± 90.82 | 148.33 ± 94.64 | 103.92 ± 95.77 | 97.65 ± 96.18 | -38.59 ± 97.92 | -48 ± 102.65 |
|  | **MAPE (%)** | 149.17 | 148.33 | 124.31 | 116.47 | 89.25 | 94.90 |
|  | **MAD (Km)** | 0.05 | 0.04 | 0.04 | 0.03 | 0.00 | 0.00 |
|  | **MAE (Km)** | 0.11 | 0.11 | 0.09 | 0.09 | 0.07 | 0.07 |
|  | **RMSE (Km)** | 0.13 | 0.13 | 0.10 | 0.10 | 0.08 | 0.08 |
|  | **ICC (95% CI)** | <0.01(-0.10 0.21) | <0.01(-0.10 0.21) | <-0.01(-0.18 0.29) | <-0.01(-0.19 0.30) | <-0.01(-0.41 0.45) | <-0.01(-0.38 0.43) |
|  |  |  |  |  |  |  |  |
| **2 Km/h** | **Mean Percentage Error ± St. Dev. (%)** | 100.63 ± 75.85 | 99.38 ± 69.04 | 90 ± 40.17 | 86.25 ± 46.17 | -11.5 ± 91 | -3.44 ± 92.72 |
|  | **MAPE (%)** | 100.63 | 99.38 | 90.00 | 86.25 | 77.50 | 71.44 |
|  | **MAD (Km)** | 0.05 | 0.04 | 0.01 | 0.03 | 0.07 | 0.06 |
|  | **MAE (Km)** | 0.10 | 0.10 | 0.09 | 0.09 | 0.08 | 0.07 |
|  | **RMSE (Km)** | 0.12 | 0.12 | 0.10 | 0.10 | 0.09 | 0.09 |
|  | **ICC (95% CI)** | <-0.01(-0.14 0.26) | <-0.01(-0.12 0.24) | <-0.01(-0.06 0.14) | <0.01(-0.08 0.17) | <-0.01(-0.51 0.49) | <0.01(-0.53 0.50) |

**Table D. Household Activities Results – Distance.**

| **HOUSEHOLD ACTIVITIES** | | **Fitbit (D)** | **Fitbit (ND)** | **Garmin (D)** | **Garmin (ND)** | **Withings (D)** | **Withings (ND)** |
| --- | --- | --- | --- | --- | --- | --- | --- |
|  |  |  |  |  |  |  |  |
| **Climb up the stairs** | **Mean Percentage Error ± St. Dev. (%)** | 60.17 ± 28.14 | 66.1 ± 26.41 | 81.39 ± 21.96 | 77.96 ± 51.37 | 62.22 ± 26.98 | 59.82 ± 32.67 |
|  | **MAPE (%)** | 60.17 | 66.10 | 81.39 | 79.66 | 63.92 | 61.52 |
|  | **MAD (Km)** | 0.01 | 0.00 | 0.00 | 0.01 | 0.00 | 0.00 |
|  | **MAE (Km)** | 0.01 | 0.01 | 0.02 | 0.02 | 0.01 | 0.01 |
|  | **RMSE (Km)** | 0.01 | 0.02 | 0.02 | 0.02 | 0.02 | 0.02 |
|  | **ICC (95% CI)** | <-0.01(-0.07 0.15) | <-0.01(-0.04 0.11) | <-0.01(-0.02 0.07) | <0.01(-0.12 0.26) | <-0.01(-0.06 0.15) | <0.01(-0.09 0.21) |
|  |  |  |  |  |  |  |  |
| **Go down the stairs** | **Mean Percentage Error ± St. Dev. (%)** | 48.3 ± 21.71 | 54.23 ± 37.1 | 77.96 ± 36.33 | 75.18 ± 34.34 | 77.96 ± 29.66 | 76.18 ± 42.77 |
|  | **MAPE (%)** | 48.30 | 57.17 | 79.34 | 76.56 | 77.96 | 77.65 |
|  | **MAD (Km)** | 0.00 | 0.01 | 0.00 | 0.00 | 0.00 | 0.01 |
|  | **MAE (Km)** | 0.01 | 0.01 | 0.02 | 0.02 | 0.02 | 0.02 |
|  | **RMSE (Km)** | 0.01 | 0.01 | 0.02 | 0.02 | 0.02 | 0.02 |
|  | **ICC (95% CI)** | <0.01(-0.06 0.13) | <-0.01(0.12 0.23) | <-0.01(-0.07 0.15) | <-0.01(-0.06 0.13) | <0.01(-0.06 0.14) | <0.01(-0.16 0.28) |
|  |  |  |  |  |  |  |  |
| **Carry a box** | **Mean Percentage Error ± St. Dev. (%)** | -1.74 ± 9.18 | -0.03 ± 9.13 | 8.7 ± 15.35 | 10.65 ± 18.78 | -7.84 ± 12.22 | -2.03 ± 15.29 |
|  | **MAPE (%)** | 6.21 | 5.80 | 12.22 | 13.76 | 11.85 | 10.63 |
|  | **MAD (Km)** | 0.02 | 0.02 | 0.01 | 0.02 | 0.03 | 0.02 |
|  | **MAE (Km)** | 0.01 | 0.01 | 0.02 | 0.03 | 0.03 | 0.02 |
|  | **RMSE (Km)** | 0.02 | 0.02 | 0.03 | 0.04 | 0.03 | 0.03 |
|  | **ICC (95% CI)** | 0.70(0.37 0.88) | 0.66(0.31 0.87) | 0.30(-0.11 0.66) | 0.14(-0.24 0.53) | 0.47(<-0.01 0.79) | 0.56(0.04 0.84) |

**Table E. Treadmill Test Results – Heart Rate.**

| **TREADMILL** | | **Fitbit (D)** | **Fitbit (ND)** | **Garmin (D)** | **Garmin (ND)** | **Philips (D)** | **Philips (ND)** |
| --- | --- | --- | --- | --- | --- | --- | --- |
|  |  |  |  |  |  |  |  |
| **1 Km/h** | **Mean Percentage Error ± St. Dev. (%)** | 3.06 ± 12.09 | 2.32 ± 14.2 | 1.71 ± 12.45 | 0.66 ± 11.73 | 4.52 ± 13.01 | 5.23 ± 10.7 |
|  | **MAPE (%)** | 9.26 | 10.60 | 9.06 | 8.33 | 8.41 | 8.26 |
|  | **MAD (bpm)** | 6.50 | 4.00 | 10.00 | 8.00 | 8.00 | 7.50 |
|  | **MAE (bpm)** | 7.36 | 8.50 | 7.07 | 6.71 | 6.29 | 6.43 |
|  | **RMSE (bpm)** | 9.34 | 10.71 | 9.18 | 9.15 | 9.50 | 8.82 |
|  | **ICC (95% CI)** | 0.81(0.51 0.94) | 0.74(0.35 0.91) | 0.86(0.61 0.95) | 0.86(0.62 0.95) | 0.84(0.58 0.94) | 0.86(0.64 0.95) |
|  |  |  |  |  |  |  |  |
| **1.5 Km/h** | **Mean Percentage Error ± St. Dev. (%)** | 4.94 ± 20.88 | 4.44 ± 19.98 | 1.79 ± 11.12 | 2.88 ± 10.4 | 7.03 ± 14.35 | 5.18 ± 14.37 |
|  | **MAPE (%)** | 12.45 | 11.12 | 8.55 | 8.19 | 11.07 | 8.92 |
|  | **MAD (bpm)** | 6.50 | 3.00 | 10.50 | 6.50 | 6.00 | 7.50 |
|  | **MAE (bpm)** | 8.64 | 7.57 | 6.93 | 6.64 | 7.79 | 6.29 |
|  | **RMSE (bpm)** | 12.16 | 11.38 | 8.93 | 8.42 | 9.80 | 9.13 |
|  | **ICC (95% CI)** | 0.69(0.26 0.70) | 0.70(0.28 0.89) | 0.83(0.55 0.94) | 0.86(0.61 0.95) | 0.78(0.46 0.93) | 0.82(0.54 0.94) |
|  |  |  |  |  |  |  |  |
| **2 Km/h** | **Mean Percentage Error ± St. Dev. (%)** | 1.67 ± 14.46 | 5.83 ± 27.19 | 2.26 ± 15.39 | 2.12 ± 14.03 | 0.52 ± 6.37 | 3.13 ± 7.12 |
|  | **MAPE (%)** | 9.65 | 13.89 | 10.22 | 10.87 | 4.54 | 6.06 |
|  | **MAD (bpm)** | 10.00 | 9.00 | 8.00 | 8.00 | 4.50 | 7.00 |
|  | **MAE (bpm)** | 8.07 | 9.57 | 7.71 | 8.50 | 3.93 | 5.00 |
|  | **RMSE (bpm)** | 11.15 | 15.53 | 10.62 | 10.26 | 5.69 | 6.60 |
|  | **ICC (95% CI)** | 0.80(0.47 0.93) | 0.52(-0.02 0.82) | 0.70(0.27 0.89) | 0.72(0.33 0.90) | 0.93(0.80 0.98) | 0.91(0.75 0.97) |

**Table F. Household Activities Results – Heart Rate.**

| **HOUSEHOLD ACTIVITIES** | | **Fitbit (D)** | **Fitbit (ND)** | **Garmin (D)** | **Garmin (ND)** | **Philips (D)** | **Philips (ND)** |
| --- | --- | --- | --- | --- | --- | --- | --- |
|  |  |  |  |  |  |  |  |
| **Climb up the stairs** | **Mean Percentage Error ± St. Dev. (%)** | -0.7 ± 7.34 | -0.45 ± 12.17 | -1.29 ± 11.12 | -3.97 ± 8.41 | 1.17 ± 6.54 | 2.35 ± 5.69 |
|  | **MAPE (%)** | 5.33 | 8.21 | 7.52 | 7.30 | 4.66 | 4.47 |
|  | **MAD (bpm)** | 6.00 | 8.00 | 9.00 | 8.00 | 11.50 | 10.50 |
|  | **MAE (bpm)** | 4.80 | 6.47 | 6.79 | 6.57 | 3.93 | 3.71 |
|  | **RMSE (bpm)** | 6.78 | 9.10 | 8.95 | 7.69 | 5.56 | 5.25 |
|  | **ICC (95% CI)** | 0.92(0.78 0.97) | 0.83(0.56 0.94) | 0.84(0.59 0.95) | 0.90(0.70 0.97) | 0.96(0.88 0.99) | 0.96(0.89 0.99) |
|  |  |  |  |  |  |  |  |
| **Go down the stairs** | **Mean Percentage Error ± St. Dev. (%)** | 1.12 ± 9.1 | 2.61 ± 13.87 | 3.05 ± 11.51 | 3.14 ± 9.38 | 2.64 ± 10.06 | 2.64 ± 10.04 |
|  | **MAPE (%)** | 5.98 | 9.19 | 7.66 | 5.65 | 6.81 | 6.04 |
|  | **MAD (bpm)** | 9.50 | 2.50 | 3.50 | 3.00 | 4.50 | 5.50 |
|  | **MAE (bpm)** | 4.79 | 6.50 | 6.14 | 4.14 | 5.08 | 4.50 |
|  | **RMSE (bpm)** | 6.38 | 8.52 | 8.36 | 6.57 | 6.95 | 6.83 |
|  | **ICC (95% CI)** | 0.91(0.75 0.97) | 0.79(0.45 0.93) | 0.83(0.54 0.94) | 0.90(0.73 0.97) | 0.86(0.60 0.96) | 0.87(0.62 0.96) |
|  |  |  |  |  |  |  |  |
| **Carry a box** | **Mean Percentage Error ± St. Dev. (%)** | -0.53 ± 7.92 | 0.99 ± 9.28 | -2.62 ± 7.75 | -2.06 ± 10.36 | 2.49 ± 12.39 | 4.33 ± 11.91 |
|  | **MAPE (%)** | 6.01 | 7.06 | 5.63 | 7.14 | 6.53 | 7.61 |
|  | **MAD (bpm)** | 6.00 | 5.00 | 7.00 | 5.00 | 8.50 | 8.00 |
|  | **MAE (bpm)** | 5.27 | 5.80 | 5.53 | 5.93 | 4.79 | 5.07 |
|  | **RMSE (bpm)** | 7.27 | 7.74 | 7.01 | 9.67 | 7.33 | 7.45 |
|  | **ICC (95% CI)** | 0.88(0.69 0.96) | 0.85(0.61 0.95) | 0.90(0.73 0.97) | 0.79(0.50 0.92) | 0.87(0.65 0.96) | 0.86(0.63 0.95) |
|  |  |  |  |  |  |  |  |
| **Dusting** | **Mean Percentage Error ± St. Dev. (%)** | -1.61 ± 9.2 | 6.05 ± 23.9 | -1.05 ± 6.89 | -0.16 ± 6.24 | -0.03 ± 5.76 | 1.06 ± 5.13 |
|  | **MAPE (%)** | 5.71 | 9.42 | 5.06 | 4.36 | 3.96 | 4.51 |
|  | **MAD (bpm)** | 10.00 | 10.00 | 5.00 | 6.00 | 11.50 | 10.50 |
|  | **MAE (bpm)** | 4.93 | 5.73 | 4.29 | 3.43 | 3.00 | 3.57 |
|  | **RMSE (bpm)** | 8.91 | 12.45 | 6.34 | 4.90 | 4.09 | 4.29 |
|  | **ICC (95% CI)** | 0.84(0.59 0.94) | 0.62(0.19 0.85) | 0.90(0.72 0.97) | 0.94(0.83 0.98) | 0.97(0.90 0.99) | 0.96(0.89 0.99) |
|  |  |  |  |  |  |  |  |
| **Rollator** | **Mean Percentage Error ± St. Dev. (%)** | -0.92 ± 4.37 | -0.42 ± 6.37 | -1.05 ± 8.8 | -2.97 ± 6.23 | 1.74 ± 7.15 | 1.69 ± 4.77 |
|  | **MAPE (%)** | 3.59 | 4.33 | 7.57 | 5.14 | 5.35 | 3.57 |
|  | **MAD (bpm)** | 9.00 | 12.50 | 10.00 | 6.00 | 8.50 | 9.50 |
|  | **MAE (bpm)** | 3.00 | 3.64 | 6.00 | 4.62 | 4.00 | 3.21 |
|  | **RMSE (bpm)** | 3.76 | 4.80 | 6.83 | 5.55 | 5.06 | 4.08 |
|  | **ICC (95% CI)** | 0.97(0.92 0.99) | 0.95(0.86 0.98) | 0.90(0.72 0.97) | 0.94(0.77 0.98) | 0.95(0.85 0.98) | 0.97(0.90 0.99) |

**Table G. Significance Test Results – Steps.**

| **Steps** | | **Fitbit (D)** | **Fitbit (ND)** | **Garmin (D)** | **Garmin (ND)** | **Philips (D)** | **Philips (ND)** | **Withings (D)** | **Withings (ND)** | **ActiGraph (A)** | **ActiGraph (W)** | **Omron** |
| --- | --- | --- | --- | --- | --- | --- | --- | --- | --- | --- | --- | --- |
|  |  |  |  |  |  |  |  |  |  |  |  |  |
| **1 Km/h** | **Vs Criterion** | **< 0.01*** | **< 0.01*** | **< 0.01** | **< 0.01** | **< 0.01*** | **< 0.01*** | **< 0.01*** | **< 0.01*** | **< 0.01*** | **< 0.01*** | **< 0.01*** |
|  | **Vs opposite arm** | 0.781 | | 0.863 | | **0.030*** | | 1.00* | | **< 0.01*** | | x |
|  |  |  |  |  |  |  |  |  |  |  |  |  |
| **1.5 Km/h** | **Vs Criterion** | 0.432 | 0.495 | **< 0.01*** | **< 0.01*** | **0.026*** | 0.438* | **< 0.01*** | **< 0.01*** | **< 0.01*** | **< 0.01*** | **< 0.01*** |
|  | **Vs opposite arm** | 0.976 | | 0.805 | | 0.673 | | 1.00* | | **< 0.01*** | | x |
|  |  |  |  |  |  |  |  |  |  |  |  |  |
| **2 Km/h** | **Vs Criterion** | 0.756* | 0.485* | **< 0.01*** | **0.014*** | 0.605* | 0.891* | **< 0.01*** | **< 0.01*** | **< 0.01*** | **< 0.01*** | **< 0.01*** |
|  | **Vs opposite arm** | 0.656 | | 0.867* | | 0.800 | | 0.583* | | **< 0.01*** | | x |
|  |  |  |  |  |  |  |  |  |  |  |  |  |
| **Climb up the stairs** | **Vs Criterion** | 0.664 | 0.640 | 0.613* | 0.492 | 0.907 | 0.694 | **< 0.01** | 0.131 | 0.060 | **< 0.01** | **< 0.01*** |
|  | **Vs opposite arm** | 0.933 | | 0.266* | | 0.891 | | 1.000 | | **0.045** | | x |
|  |  |  |  |  |  |  |  |  |  |  |  |  |
| **Go down the stairs** | **Vs Criterion** | **0.011** | 0.058 | 0.717* | 0.641* | 0.076 | **< 0.01** | **0.017** | **0.020** | **0.019*** | **< 0.01** | **< 0.01*** |
|  | **Vs opposite arm** | 0.714 | | 0.750* | | 0.269 | | 0.401 | | 0.153* | | x |
|  |  |  |  |  |  |  |  |  |  |  |  |  |
| **Carry a box** | **Vs Criterion** | 0.513 | 0.576 | 0.957 | 0.821 | **0.049*** | 0.331* | **< 0.01** | **0.022** | 0.523 | **< 0.01** | 0.250 |
|  | **Vs opposite arm** | 0.93 | | 0.85 | | 0.556* | | 0.539 | | **< 0.01** | | x |
|  |  |  |  |  |  |  |  |  |  |  |  |  |
| **Dusting** | **Vs Criterion** | 0.167 | **< 0.01*** | 0.247* | **< 0.01** | 0.093 | **0.012** | 0.972 | **< 0.01*** | **< 0.01*** | **< 0.01*** | **< 0.01*** |
|  | **Vs opposite arm** | **< 0.01*** | | **< 0.01*** | | **< 0.01*** | | **< 0.01*** | | 0.265 | | x |
|  |  |  |  |  |  |  |  |  |  |  |  |  |
| **Rollator** | **Vs Criterion** | **< 0.01*** | **< 0.01*** | **< 0.01*** | **< 0.01*** | **< 0.01*** | **< 0.01*** | **< 0.01*** | **< 0.01*** | **< 0.01*** | **< 0.01*** | **< 0.01*** |
|  | **Vs opposite arm** | 0.123* | | 1.00* | | 0.394* | | 1.00* | | **< 0.01*** | | x |

p-value is indicated with an asterisk if it has been estimated through a Wilcoxon signed-rank test, while it is presented in bold if lower than the 0.05 significance threshold.

**Table H. Significance Test Results – Distance.**

| **Distance** | | **Fitbit (D)** | **Fitbit (ND)** | **Garmin (D)** | **Garmin (ND)** | **Withings (D)** | **Withings (ND)** |
| --- | --- | --- | --- | --- | --- | --- | --- |
|  |  |  |  |  |  |  |  |
| **1 Km/h** | **Vs Criterion** | **< 0.01*** | **< 0.01*** | 0.052* | 0.065* | 0.195* | 0.155* |
|  | **Vs opposite arm** | 0.823 | | 0.805 | | 0.844* | |
|  |  |  |  |  |  |  |  |
| **1.5 Km/h** | **Vs Criterion** | **< 0.01*** | **< 0.01*** | **< 0.01*** | **< 0.01*** | **0.028*** | **0.011** |
|  | **Vs opposite arm** | 0.794 | | 0.850 | | 0.820* | |
|  |  |  |  |  |  |  |  |
| **2 Km/h** | **Vs Criterion** | **< 0.01*** | **< 0.01*** | **< 0.01*** | **< 0.01*** | 0.287* | 0.716* |
|  | **Vs opposite arm** | 0.961 | | 0.682* | | 0.505* | |
|  |  |  |  |  |  |  |  |
| **Climb up the stairs** | **Vs Criterion** | **< 0.01*** | **< 0.01*** | **< 0.01*** | **< 0.01** | **< 0.01*** | **< 0.01*** |
|  | **Vs opposite arm** | 0.237* | | 0.748* | | 0.859* | |
|  |  |  |  |  |  |  |  |
| **Go down the stairs** | **Vs Criterion** | **< 0.01*** | **< 0.01*** | **< 0.01*** | **< 0.01*** | **< 0.01** | **< 0.01*** |
|  | **Vs opposite arm** | 0.877* | | 0.380* | | 0.856* | |
|  |  |  |  |  |  |  |  |
| **Carry a box** | **Vs Criterion** | 0.145* | 0.945 | 0.074 | **0.048** | 0.145 | 0.862 |
|  | **Vs opposite arm** | 0.088* | | 0.725 | | 0.342 | |

p-value is indicated with an asterisk if it has been estimated through a Wilcoxon signed-rank test, while it is presented in bold if lower than the 0.05 significance threshold.

**Table I. Significance Test Results – Heart Rate.**

| **Heart rate** | | **Fitbit (D)** | **Fitbit (ND)** | **Garmin (D)** | **Garmin (ND)** | **Philips (D)** | **Philips (ND)** |
| --- | --- | --- | --- | --- | --- | --- | --- |
|  |  |  |  |  |  |  |  |
| **1 Km/h** | **Vs Criterion** | 0.816 | 0.930 | 0.940 | 0.863 | 0.671 | 0.595 |
|  | **Vs opposite arm** | 0.865 | | 0.903 | | 0.910 | |
|  |  |  |  |  |  |  |  |
| **1.5 Km/h** | **Vs Criterion** | 0.744 | 0.800 | 0.952 | 0.821 | 0.114* | 0.875* |
|  | **Vs opposite arm** | 0.917 | | 0.846 | | 0.374* | |
|  |  |  |  |  |  |  |  |
| **2 Km/h** | **Vs Criterion** | 0.992 | 0.815 | 0.978 | 0.968 | 0.951 | 0.681 |
|  | **Vs opposite arm** | 0.801 | | 0.986 | | 0.735 | |
|  |  |  |  |  |  |  |  |
| **Climb up the stairs** | **Vs Criterion** | 0.864 | 0.752 | 0.734 | 0.586 | 0.946 | 0.847 |
|  | **Vs opposite arm** | 0.891 | | 0.799 | | 0.898 | |
|  |  |  |  |  |  |  |  |
| **Go down the stairs** | **Vs Criterion** | 0.836 | 0.853 | 0.864 | 0.672* | 0.831 | 0.787 |
|  | **Vs opposite arm** | 0.953 | | 0.824* | | 0.953 | |
|  |  |  |  |  |  |  |  |
| **Carry a box** | **Vs Criterion** | 0.857 | 0.990 | 0.635 | 0.623 | 0.867 | 0.679 |
|  | **Vs opposite arm** | 0.850 | | 1.000 | | 0.789 | |
|  |  |  |  |  |  |  |  |
| **Dusting** | **Vs Criterion** | 0.764 | 0.634 | 0.791 | 0.918 | 0.962 | 0.907 |
|  | **Vs opposite arm** | 0.409 | | 0.861 | | 0.867 | |
|  |  |  |  |  |  |  |  |
| **Rollator** | **Vs Criterion** | 0.888 | 0.915 | 0.802 | 0.632 | 0.866 | 0.842 |
|  | **Vs opposite arm** | 0.971 | | 0.808 | | 0.971 | |

p-value is indicated with an asterisk if it has been estimated through a Wilcoxon signed-rank test, while it is presented in bold if lower than the 0.05 significance threshold.

**Table J. Bland-Altman Plots Summary – Steps.**

| **ME ± St. Dev. (95% LoA) (Steps)** | | **Fitbit (D)** | **Fitbit (ND)** | **Garmin (D)** | **Garmin (ND)** | **Philips (D)** | **Philips (ND)** |
| --- | --- | --- | --- | --- | --- | --- | --- |
|  |  |  |  |  |  |  |  |
| **1 Km/h** | **Vs Criterion** | -101.06 ± 65.5 (-229.44, 27.32) | -91.35 ± 79.6 (-247.37, 64.67) | -118.29 ± 74.47 (-264.25, 27.67) | -111.41 ± 67.6 (-243.91, 21.09) | -110 ± 91.31 (-288.97, 68.97) | -82.65 ± 102.6 (-283.75, 118.45) |
|  | **Vs opposite arm** | -9.71 ± 89.45 (-185.02, 165.61 ) | | -6.88 ± 75.22 (-154.32, 140.56 ) | | -27.35 ± 51.36 (-128.01, 73.31 ) | |
|  |  |  |  |  |  |  |  |
| **1.5 Km/h** | **Vs Criterion** | 20.5 ± 85.77 (-147.61, 188.61) | 19.5 ± 86.81 (-150.65, 189.65) | -50.71 ± 46.43 (-141.71, 40.29) | -58.53 ± 58.75 (-173.68, 56.62) | -44.56 ± 89.32 (-219.63, 130.51) | -29.19 ± 83.43 (-192.71, 134.33) |
|  | **Vs opposite arm** | 1 ± 97.25 (-189.61, 191.61) | | 7.82 ± 57.92 (-105.7 , 121.34) | | -15.38 ± 92.87 (-197.41, 166.65) | |
|  |  |  |  |  |  |  |  |
| **2 Km/h** | **Vs Criterion** | 5.69 ± 98.93 (-188.21, 199.59) | 19.19 ± 92.01 (-161.15, 199.53) | -18.63 ± 15.61 (-49.23, 11.97) | -19.75 ± 34.35 (-87.08, 47.58) | 8.93 ± 66.16 (-120.74, 138.6) | 6.31 ± 51.17 (-93.98, 106.6) |
|  | **Vs opposite arm** | -13.5 ± 102.75 (-214.89, 187.89) | | 1.13 ± 26.75 (-51.3, 53.56) | | -6.5 ± 60.69 (-125.45, 112.45) | |
|  |  |  |  |  |  |  |  |
| **Climb up the stairs** | **Vs Criterion** | -0.73 ± 6.37 (-13.22, 11.76) | -0.93 ± 6.46 (-13.59, 11.73) | -3.23 ± 12.23 (-27.2, 20.74) | -1.92 ± 8.2 (-17.99, 14.15) | 0.43 ± 13.64 (-26.3, 27.16) | -0.21 ± 11.12 (-22.01, 21.59) |
|  | **Vs opposite arm** | 0.2 ± 6.09 (-11.74, 12.14) | | -1.31 ± 11.44 (-23.73, 21.11) | | 0.64 ± 8.83 (-16.67, 17.95) | |
|  |  |  |  |  |  |  |  |
| **Go down the stairs** | **Vs Criterion** | -4.8 ± 7.06 (-18.64, 9.04) | -6 ± 11.95 (-29.42, 17.42) | -1.69 ± 8.58 (-18.51, 15.13) | -2.06 ± 10.64 (-22.91, 18.79) | -5.57 ± 10.24 (-25.64, 14.5) | -9.36 ± 8.54 (-26.1, 7.38) |
|  | **Vs opposite arm** | 1.2 ± 9.84 (-18.09, 20.49) | | 0.38 ± 9.98 (-19.18, 19.94) | | 3.79 ± 14.48 (-24.59, 32.17) | |
|  |  |  |  |  |  |  |  |
| **Carry a box** | **Vs Criterion** | -7.67 ± 11.91 (-31.01, 15.67) | -6.56 ± 13.76 (-33.53 , 20.41) | -0.65 ± 37.73 (-74.6, 73.3) | -2.76 ± 38.48 (-78.18, 72.66) | -4.44 ± 48.37 (-99.25, 90.37) | 3.94 ± 52.67 (-99.29, 107.17) |
|  | **Vs opposite arm** | -1.11 ± 6.52 (-13.89, 11.67) | | 2.12 ± 9.51 (-16.52, 20.76) | | -8.39 ± 25.11 (-57.61, 40.83) | |
|  |  |  |  |  |  |  |  |
| **Dusting** | **Vs Criterion** | 22.71 ± 71.23 (-116.9, 162.32) | -77.06 ± 38.51 (-152.54, -1.58) | -19.4 ± 62.5 (-141.9, 103.1) | -66.6 ± 32.63 (-130.55, -2.65) | 29.12 ± 59.69 (-87.87, 146.11) | -46 ± 60.3 (-164.19, 72.19) |
|  | **Vs opposite arm** | 99.76 ± 63.32 (-24.35, 223.87) | | 47.2 ± 63.79 (-77.83, 172.23) | | 75.12 ± 84.56 (-90.62, 240.86) | |
|  |  |  |  |  |  |  |  |
| **Rollator** | **Vs Criterion** | -159.25 ± 68.38 (-293.27, -25.23) | -104.31 ± 103.24 (-306.66, 98.04) | -219.06 ± 36.38 (-290.36, -147.76) | -219 ± 36.21 (-289.97, -148.03) | -191.56 ± 50 (-289.56, -93.56) | -193.44 ± 50.01 (-291.46, -95.42) |
|  | **Vs opposite arm** | -54.94 ± 113.44 (-277.28 , 167.4) | | -0.06 ± 0.93 (-1.88 , 1.76) | | 1.89 ± 11.16 (-19.98, 23.76) | |

**Table J. Bland-Altman Plots Summary – Steps (continued).**

| **ME ± St. Dev. (95% LoA) (Steps)** | | **Withings (D)** | **Withings (ND)** | **ActiGraph (A)** | **ActiGraph (W)** | **Omron** |
| --- | --- | --- | --- | --- | --- | --- |
|  |  |  |  |  |  |  |
| **1 Km/h** | **Vs Criterion** | -200.41 ± 49.77 (-297.96, -102.86) | -201.59 ± 56.59 (-312.51, -90.67) | -107.85 ± 62.48 (-230.31, 14.61) | -207.91 ± 63.46 (-332.29, -83.53) | -177.76 ± 72.9 (-320.64, -34.88) |
|  | **Vs opposite arm** | 1.18 ± 57.88 (-112.27, 114.62 ) | | 100.06 ± 94.17 (-84.51, 284.63) | | x |
|  |  |  |  |  |  |  |
| **1.5 Km/h** | **Vs Criterion** | -181.18 ± 74.71 (-327.61, -34.75) | -188.59 ± 57.18 (-300.66, -76.52) | -69.21 ± 60.66 (-188.1, 49.68) | -183.65 ± 62.84 (-306.82, -60.48) | -140.59 ± 82.35 (-302, 20.82) |
|  | **Vs opposite arm** | 7.41 ± 47.78 (-86.24, 101.06) | | 114.44 ± 84.66 (-51.49, 280.37) | | x |
|  |  |  |  |  |  |  |
| **2 Km/h** | **Vs Criterion** | -155.31 ± 96.81 (-345.06, 34.44) | -144.56 ± 83.91 (-309.02, 19.9) | -19.47 ± 29.68 (-77.64, 38.7) | -159.63 ± 77.37 (-311.28, -7.98) | -84.94 ± 85.43 (-252.38 , 82.5) |
|  | **Vs opposite arm** | -10.75 ± 69.36 (-146.7, 125.2) | | 139.68 ± 78.71 (-14.59, 293.95) | | x |
|  |  |  |  |  |  |  |
| **Climb up the stairs** | **Vs Criterion** | -5.69 ± 6.97 (-19.35, 7.97) | -5.69 ± 12.04 (-29.29, 17.91) | -1.68 ± 4.09 (-9.7, 6.34) | -4.39 ± 7.4 (-18.89 , 10.11) | -2.87 ± 5.35 (-13.36, 7.62) |
|  | **Vs opposite arm** | 0 ± 13.8 (-27.05, 27.05) | | 2.57 ± 6.66 (-10.48, 15.62) | | x |
|  |  |  |  |  |  |  |
| **Go down the stairs** | **Vs Criterion** | -4.73 ± 5.65 (-15.8, 6.34) | -7.47 ± 11.73 (-30.46 , 15.52) | -16.55 ± 27.58 (-70.61 , 37.51) | -5.23 ± 4.95 (-14.93, 4.47) | -2.1 ± 3.44 (-8.84 , 4.64) |
|  | **Vs opposite arm** | 2.73 ± 10.4 (-17.65, 23.11) | | -12.21 ± 29.17 (-69.38, 44.96) | | x |
|  |  |  |  |  |  |  |
| **Carry a box** | **Vs Criterion** | -48.14 ± 41.7 (-129.87, 33.59) | -39 ± 42.53 (-122.36, 44.36) | -7.06 ± 28.66 (-63.23, 49.11) | -39.06 ± 48.4 (-133.92, 55.8) | -9.63 ± 31.03 (-70.45, 51.19) |
|  | **Vs opposite arm** | -9.14 ± 34.32 (-76.41, 58.13) | | 33.1 ± 44.69 (-54.49, 120.69) | | x |
|  |  |  |  |  |  |  |
| **Dusting** | **Vs Criterion** | 0.6 ± 66.01 (-128.78, 129.98) | -87.4 ± 23.13 (-132.73, -42.07) | -80.58 ± 23.97 (-127.56, -33.6) | -80.64 ± 24.7 (-129.05, -32.23) | -82.27 ± 23.18 (-127.7, -36.84) |
|  | **Vs opposite arm** | 88 ± 63.29 (-36.05, 212.05) | | 0.06 ± 23.22 (-45.45, 45.57) | | x |
|  |  |  |  |  |  |  |
| **Rollator** | **Vs Criterion** | -212.81 ± 39.49 (-290.21, -135.41) | -212.81 ± 39.52 (-290.27, -135.35) | -83.3 ± 60.54 (-201.96, 35.36) | -170.67 ± 33.11 (-235.57, -105.77) | -111.52 ± 55.72 (-220.73, -2.31) |
|  | **Vs opposite arm** | 0 ± 2.19 (-4.29 , 4.29) | | 87.36 ± 68.54 (-46.98, 221.7) | | x |

**Table K. Bland-Altman Plots Summary – Distance.**

| **ME ± St. Dev. (95% LoA) (Distance)** | | **Fitbit (D)** | **Fitbit (ND)** | **Garmin (D)** | **Garmin (ND)** | **Withings (D)** | **Withings (ND)** |
| --- | --- | --- | --- | --- | --- | --- | --- |
|  |  |  |  |  |  |  |  |
| **1 Km/h** | **Vs Criterion** | 0.06 ± 0.06 (-0.06, 0.18) | 0.06 ± 0.07 (-0.08, 0.2) | 0.04 ± 0.08 (-0.12, 0.2) | 0.05 ± 0.09 (-0.13, 0.23) | -0.01 ± 0.07 (-0.15, 0.13) | -0.01 ± 0.08 (-0.17, 0.15) |
|  | **Vs opposite arm** | -0.01 ± 0.06 (-0.13, 0.11) | | -0.01 ± 0.06 (-0.13, 0.11) | | 0 ± 0.05 (-0.1, 0.1) | |
|  |  |  |  |  |  |  |  |
| **1.5 Km/h** | **Vs Criterion** | 0.11 ± 0.07 (-0.03, 0.25) | 0.11 ± 0.07 (-0.03, 0.25) | 0.08 ± 0.07 (-0.06, 0.22) | 0.07 ± 0.07 (-0.07, 0.21) | -0.03 ± 0.07 (-0.17, 0.11) | -0.04 ± 0.08 (-0.2, 0.12) |
|  | **Vs opposite arm** | 0 ± 0.07 (-0.14, 0.14) | | 0 ± 0.04 (-0.08, 0.08) | | 0.01 ± 0.04 (-0.07, 0.09) | |
|  |  |  |  |  |  |  |  |
| **2 Km/h** | **Vs Criterion** | 0.1 ± 0.08 (-0.06, 0.26) | 0.1 ± 0.07 (-0.04, 0.24) | 0.09 ± 0.04 (0.01, 0.17) | 0.09 ± 0.05 (-0.01, 0.19) | -0.01 ± 0.09 (-0.19, 0.17) | 0 ± 0.09 (-0.18, 0.18) |
|  | **Vs opposite arm** | 0 ± 0.1 (-0.2, 0.2) | | 0 ± 0.02 (-0.04, 0.04) | | -0.01 ± 0.06 (-0.13, 0.11) | |
|  |  |  |  |  |  |  |  |
| **Climb up the stairs** | **Vs Criterion** | 0.01 ± 0.01 (-0.01, 0.03) | 0.02 ± 0.01 (0, 0.04) | 0.02 ± 0 (0.02, 0.02) | 0.02 ± 0.01 (0, 0.04) | 0.01 ± 0.01 (-0.01, 0.03) | 0.01 ± 0.01 (-0.01, 0.03) |
|  | **Vs opposite arm** | 0 ± 0.01 (-0.02, 0.02) | | 0 ± 0.01 (-0.02, 0.02) | | 0 ± 0.01 (-0.02, 0.02) | |
|  |  |  |  |  |  |  |  |
| **Go down the stairs** | **Vs Criterion** | 0.01 ± 0.01 (-0.01, 0.03) | 0.01 ± 0.01 (-0.01, 0.03) | 0.02 ± 0.01 (0, 0.04) | 0.02 ± 0.01 (0, 0.04) | 0.02 ± 0.01 (0, 0.04) | 0.02 ± 0.01 (0, 0.04) |
|  | **Vs opposite arm** | 0 ± 0.01 (-0.02, 0.02) | | 0 ± 0.01 (-0.02, 0.02) | | 0 ± 0.02 (-0.04, 0.04) | |
|  |  |  |  |  |  |  |  |
| **Carry a box** | **Vs Criterion** | 0 ± 0.02 (-0.04, 0.04) | 0 ± 0.02 (-0.04, 0.04) | 0.02 ± 0.03 (-0.04, 0.08) | 0.02 ± 0.03 (-0.04, 0.08) | -0.02 ± 0.03 (-0.08, 0.04) | 0 ± 0.03 (-0.06, 0.06) |
|  | **Vs opposite arm** | 0 ± 0.01 (-0.02, 0.02) | | 0 ± 0.01 (-0.02, 0.02) | | -0.01 ± 0.03 (-0.07, 0.05) | |

**Table L. Bland-Altman Plots Summary – Heart Rate.**

| **ME ± St. Dev. (95% LoA) (Heart rate)** | | **Fitbit (D)** | **Fitbit (ND)** | **Garmin (D)** | **Garmin (ND)** | **Philips (D)** | **Philips (ND)** |
| --- | --- | --- | --- | --- | --- | --- | --- |
|  |  |  |  |  |  |  |  |
| **1 Km/h** | **Vs Criterion** | 1.36 ± 9.59 (-17.44, 20.16) | 0.5 ± 11.11 (-21.28, 22.28) | -0.5 ± 9.52 (-19.16, 18.16) | -1.14 ± 9.42 (-19.6, 17.32) | 2.7 ± 9.45 (-15.82, 21.22) | 3.43 ± 8.43 (-13.09, 19.95) |
|  | **Vs opposite arm** | 0.86 ± 2.91 (-4.84, 6.56) | | 0.64 ± 3.48 (-6.18, 7.46) | | -0.71 ± 4.25 (-9.04, 7.62) | |
|  |  |  |  |  |  |  |  |
| **1.5 Km/h** | **Vs Criterion** | 1.93 ± 12.46 (-22.49, 26.35) | 1.43 ± 11.72 (-21.54, 24.4) | 0.36 ± 9.26 (-17.79, 18.51) | 1.36 ± 8.63 (-15.55, 18.27) | 4.21 ± 9.18 (-13.78, 22.2) | 3 ± 8.95 (-14.54, 20.54) |
|  | **Vs opposite arm** | 0.5 ± 5.06 (-9.42, 10.42) | | -1 ± 3.44 (-7.74, 5.74) | | 1.21 ± 3.98 (-6.59, 9.01) | |
|  |  |  |  |  |  |  |  |
| **2 Km/h** | **Vs Criterion** | 0.07 ± 11.57 (-22.61, 22.75) | 1.43 ± 16.05 (-30.03, 32.89) | 0.14 ± 11.02 (-21.46, 21.74) | 0.21 ± 10.65 (-20.66, 21.08) | 0.36 ± 5.89 (-11.18, 11.9) | 2.43 ± 6.37 (-10.06, 14.92) |
|  | **Vs opposite arm** | -1.36 ± 13.29 (-27.41, 24.69) | | -0.07 ± 6.27 (-12.36, 12.22) | | -2.07 ± 3.45 (-8.83, 4.69) | |
|  |  |  |  |  |  |  |  |
| **Climb up the stairs** | **Vs Criterion** | -1.07 ± 6.93 (-14.65, 12.51) | -1.8 ± 9.24 (-19.91, 16.31) | -2.07 ± 9.03 (-19.77, 15.63) | -3.57 ± 7.07 (-17.43, 10.29) | 0.5 ± 5.75 (-10.77, 11.77) | 1.43 ± 5.24 (-8.84, 11.7) |
|  | **Vs opposite arm** | 0.73 ± 10.45 (-19.75, 21.21) | | 1.5 ± 7.77 (-13.73, 16.73) | | -0.93 ± 2.97 (-6.75, 4.89) | |
|  |  |  |  |  |  |  |  |
| **Go down the stairs** | **Vs Criterion** | 0.5 ± 6.6 (-12.44, 13.44) | 0.5 ± 8.83 (-16.81, 17.81) | 1.29 ± 8.57 (-15.51, 18.09) | 1.71 ± 6.58 (-11.19, 14.61) | 1.75 ± 7.02 (-12.01, 15.51) | 1.83 ± 6.87 (-11.64, 15.3) |
|  | **Vs opposite arm** | 0 ± 7.84 (-15.37, 15.37) | | -0.43 ± 4.2 (-8.66, 7.8) | | -0.08 ± 3.82 (-7.57, 7.41) | |
|  |  |  |  |  |  |  |  |
| **Carry a box** | **Vs Criterion** | -1 ± 7.45 (-15.6, 13.6) | -0.07 ± 8.01 (-15.77, 15.63) | -2.73 ± 6.68 (-15.82, 10.36) | -2.73 ± 9.6 (-21.55, 16.09) | 0.93 ± 7.55 (-13.87, 15.73) | 2.21 ± 7.38 (-12.25, 16.67) |
|  | **Vs opposite arm** | -0.93 ± 4.57 (-9.89, 8.03) | | 0 ± 5.26 (-10.31, 10.31) | | -1.29 ± 3.45 (-8.05, 5.47) | |
|  |  |  |  |  |  |  |  |
| **Dusting** | **Vs Criterion** | -1.73 ± 9.04 (-19.45, 15.99) | 2.53 ± 12.62 (-22.21, 27.27) | -1.43 ± 6.41 (-13.99, 11.13) | -0.57 ± 5.05 (-10.47, 9.33) | -0.29 ± 4.23 (-8.58, 8) | 0.71 ± 4.39 (-7.89, 9.31) |
|  | **Vs opposite arm** | -4.27 ± 14.67 (-33.02, 24.48) | | -0.86 ± 3.8 (-8.31, 6.59) | | -1 ± 4.15 (-9.13, 7.13) | |
|  |  |  |  |  |  |  |  |
| **Rollator** | **Vs Criterion** | -0.86 ± 3.8 (-8.31, 6.59) | -0.64 ± 5.14 (-10.71, 9.43) | -1.54 ± 6.92 (-15.1, 12.02) | -2.92 ± 4.89 (-12.5, 6.66) | 1 ± 5.14 (-9.07, 11.07) | 1.21 ± 4.04 (-6.71, 9.13) |
|  | **Vs opposite arm** | -0.21 ± 3.79 (-7.64, 7.22) | | 1.38 ± 4.87 (-8.17, 10.93) | | -0.21 ± 3.58 (-7.23, 6.81) | |
